# Exploring the Potential of Heteroatom-Doped Graphene Nanoribbons as a Catalyst for Oxygen Reduction

**DOI:** 10.3390/nano13212831

**Published:** 2023-10-26

**Authors:** Eduardo S. F. Cardoso, Guilherme V. Fortunato, Clauber D. Rodrigues, Marcos R. V. Lanza, Gilberto Maia

**Affiliations:** 1 Institute of Chemistry, Federal University of Mato Grosso do Sul, Av. Senador Filinto Muller 1555, Campo Grande 79074-460, MS, Brazil; eduardosafreca@gmail.com; 2 São Carlos Institute of Chemistry, University of São Paulo, Avenida Trabalhador São-Carlense 400, São Carlos 13566-590, SP, Brazil; g.fortunato@usp.br (G.V.F.); marcoslanza@usp.br (M.R.V.L.); 3 Campus Glória de Dourados, State University of Mato Grosso do Sul, Rua Rogério Luis Rodrigues s/n, Glória de Dourados 79730-000, MS, Brazil; clauber@uems.br

**Keywords:** oxygen-reduction reaction, hydrogen peroxide production, graphene nanoribbon, heteroatom doping, functional groups

## Abstract

In this study, we created a series of N, S, and P-doped and co-doped carbon catalysts using a single graphene nanoribbon (GNR) matrix and thoroughly evaluated the impact of doping on ORR activity and selectivity in acidic, neutral, and alkaline conditions. The results obtained showed no significant changes in the GNR structure after the doping process, though changes were observed in the surface chemistry in view of the heteroatom insertion and oxygen depletion. Of all the dopants investigated, nitrogen (mainly in the form of pyrrolic-N and graphitic-N) was the most easily inserted and detected in the carbon matrix. The electrochemical analyses conducted showed that doping impacted the performance of the catalyst in ORR through changes in the chemical composition of the catalyst, as well as in the double-layer capacitance and electrochemically accessible surface area. In terms of selectivity, GNR doped with phosphorus and sulfur favored the 2e^−^ ORR pathway, while nitrogen favored the 4e^−^ ORR pathway. These findings can provide useful insights into the design of more efficient and versatile catalytic materials for ORR in different electrolyte solutions, based on functionalized carbon.

## 1. Introduction

Graphene is a single-layer, 2D material made of carbon atoms under a crystalline arrangement [1]; owing to its large surface area and ease of functionalization through heteroatom doping, this carbon material is extremely valued for application in chemical reactions. In addition, its low density, high conductivity, chemical stability, and resistance to corrosion make graphene an efficient, durable material for catalytic applications [1,2,3].

Graphene comes in a variety of forms, and each form is distinctively characterized by its peculiar structural, electrical, and optical properties. Graphene nanoribbons (GNRs) are a special class of graphene that has gained traction among researchers; GNRs have been widely applied in catalytic reactions/processes due to their unique physical–chemical properties [4]. GNRs are thin strips of graphene—measuring just a few nanometers wide (commonly described as 1D material), which can exhibit a metallic or semi-conductor characteristic depending on their width and edge structure [2,4,5]. The suitable properties of GNRs make them useful for application in electronics, sensing devices, and energy storage [2,4].

GNRs can be synthesized through a wide range of methods including chemical vapor deposition [2], patterning with lithography techniques [6], or through chemical oxidation of carbon nanotubes [5,7]. GNRs have been successfully applied alone or in combination with other materials in different electrolyte solutions to catalyze several electrochemical reactions including water splitting [8], CO_2_ reduction reaction [9,10], and oxygen reduction reaction (ORR) [7,11,12,13]. Incorporating heteroatoms like nitrogen, sulfur, or oxygen into carbon materials—a mechanism referred to as doping, provides us with considerable benefits, as it leads to the development of carbon materials with suitable properties that are highly efficient for catalytic applications [14,15]. Doping alters the electronic structure of the carbon material, and this can effectively boost its ORR catalytic activity in acidic, neutral, and alkaline media. For instance, incorporating nitrogen into graphene leads to the development of pyridinic nitrogen—a material with high electron density, which increases the catalytic activity of graphene in ORR, particularly in alkaline environments [15]. Similarly, adding sulfur or oxygen to graphene creates defects or dopants, which enhance the catalytic activity and selectivity of the material [15,16,17]. However, the ORR activity and selectivity exhibited by heteroatom-doped carbon catalysts can undergo dramatic variations depending on the pH of the electrolyte [18], yet these phenomena remain inadequately understood.

Certain doped carbon materials showcase heightened activity in either acidic or alkaline conditions, while others demonstrate remarkable performance across a broad pH range [18,19,20,21,22]. These differences can be attributed to divergent behaviors of the reactants (i.e., protons or hydroxide ions and their availabilities) and intermediates. Furthermore, heteroatom-doped carbon catalysts may present a multitude of enriched reversible redox couples on their surfaces, serving as conduits for charge transfer to adsorbed oxygen [19]. The electrochemical activity or inactivity of these redox couples toward the ORR is contingent upon the specific electrolyte employed [19]. Doping may also help improve the stability and durability of carbon materials, as is the case of nitrogen-doped carbon materials, which exhibit greater resistance to oxidative degradation and longer lifetime when applied in catalytic reactions/processes [23].

Recently, considerable attention has been devoted toward creating nitrogen, oxygen, sulfur, and phosphorus-doped graphene structures targeted at enhancing the electrocatalytic performance of the carbon materials, especially in ORR processes. Regarding the development of ORR electrocatalytic materials, although a plethora of studies have been reported in the literature, the study conducted by Xiang et al. [24] appears to be outstanding. Xiang et al. [24] found that N-doped GNRs, produced through the oxidative unzipping of CNTs and N-doping with urea, and its abundant edges have a synergistic effect on the ORR process through the four-electron pathway; these authors also pointed out that pyridinic and graphitic-N are the main contributors when it comes to catalyzing the ORR process. 

Based on the application of the hard-templating method, Dong et al. [25] produced porous graphene foam doped with B, N, and P, which effectively enhanced ORR performance in comparison with graphene foam doped with single or dual elements. Han and Chen [26] showed that keeping the N proportion more than twice as high as the proportion of P in graphene co-doped with P and N (G-PN_3_) improved the catalytic activity in ORR and selectivity under the four electron mechanism. Zhao et al. [27] found that doping ordered mesoporous carbon (OMC) first with P and later with N led to an increase in the graphitic-N ratio and improved the catalytic activity in ORR. Yang et al. [28] developed and applied edge-rich graphene nanoribbon co-doped with N and S via thermal annealing in ORR, which led to significant improvements in catalytic activity. Wang et al. [29] used a 3-D-structured carbon nanotube/GNRs co-doped with N and S from the thermal treatment of thiourea, which effectively catalyzed a four-electron ORR process. Kan et al. [30] synthesized carbon nanospheres doped with N and N and S (NCSs and NSCSs) through melanosome pyrolysis with NSCS; the application of this carbon-doped material resulted in better catalytic activity in ORR aimed at water production. 

Li et al. [31] synthesized 3-D-reduced graphene oxide co-doped with N and S (NS-3DrGO) through pyrolysis; the application of this material resulted in better ORR activity with the successful transfer of four electrons. Zhai et al. [32] produced graphene oxide doped with S by using DMSO as a solvent and S precursor and the application of the solvothermal method; the authors successfully applied the S-RGO in ORR with good results. Yazdi et al. [33] produced helical CN_x_-GNRs co-doped with N and S (CNx/CS_x_-GNRs) through annealing, where the application of the material resulted in efficient ORR activity and four-electron selectivity. The authors of the aforementioned study attributed the efficient ORR activity to the synergistic effects derived from the co-doping of nitrogen/sulfur and to the helical unzipping mechanism, which gave rise to graphene nanoribbons with multifaceted structure; according to the authors, the S co-doping mechanism led to an increase in pyridinic-N groups in the GNR structure—these groups constitute the main active sites that helped enhance ORR activity on the edges [33]. In another related study, through annealing, Wang et al. [34] synthesized a co-doped N and P 3-D structured GNR/CNT composite through annealing; the application of the synthesized material as a ORR catalyst resulted in a satisfactory performance. 

Tammeveski and co-workers [35] employed both wet and dry ball-milling methods to produce sulfur and nitrogen co-doped graphene-based catalysts. These catalysts exhibited a significant presence of pyridinic N in both cases, with the catalysts produced by dry ball-milling being more suitable for facilitating a complete 4e^−^ oxygen reduction. Additionally, they synthesized silicon carbide-derived carbon (SiCDC) doped with nitrogen and phosphorus moieties using a ball-milling method, which improved the 4e^−^ ORR pathway by incorporating active sites derived from the nitrogen and phosphorus moieties [36]. Furthermore, they successfully synthesized N,P-doped SiCDC and CNT with hierarchical pore structures via a ball-milling method, leading to further improvements in the 4e^−^ ORR pathway [37]. Dey and co-workers [38] synthesized a triazine-based covalent organic polymer (Trz-COP) metal-free electrocatalyst with dual-active sites. They also employed a polymer-assisted electrophoretic exfoliation method on graphite to produce graphene–polypyrrole (G-PPy) in a dilute acidic medium, followed by a high-temperature treatment to incorporate N atoms into the graphene matrix. This resulted in the formation of an N-doped graphene-PPy (NG-PPy) metal-free catalyst [39]. Additionally, they synthesized a bis(terpyridine) (hexadentate chelating ligand) with Fe, promoting the formation of FeN_x_/C active sites (Fe-N/C_(H,P)_ electrocatalyst) [40]. These catalysts demonstrated efficient ORR electrocatalysis, predominantly resulting in the production of water.

Based on the above considerations, it is clear that doping carbon materials with heteroatoms, such as nitrogen, sulfur, oxygen, and phosphorus, leads to the development of carbon materials with suitable properties that have great potential for catalytic applications; strangely, though, this technique has still not been fully explored in the literature, especially considering the specific benefits that can be derived from the doping mechanism, depending on the heteroatom, medium, and type of reaction involved. Finding an electrocatalyst that can efficiently produce water or hydrogen peroxide through oxygen reduction reaction (ORR) in different electrolyte solutions remains a daunting challenge today. In the present work, we developed and characterized a series of N, S, and P-doped and co-doped carbon catalysts using the same GNR matrix and thoroughly evaluated the impact of doping on the catalytic activity in ORR and selectivity under acidic, neutral, and alkaline conditions using the rotating ring-disk electrode (RRDE) technique. The results obtained from the characterization of the proposed materials showed that the heteroatom doping process did not significantly alter the GNR structure, though it altered the surface chemistry, which was caused by the insertion of the heteroatoms and oxygen depletion. Among the dopants investigated in this study, N was the most easily inserted and detected in the carbon matrix. The electrochemical analyses conducted revealed that heteroatom doping or co-doping and residual oxygen levels influenced the physicochemical properties of the catalyst, as well as the ORR activity and selectivity, which varied with changes in the electrolyte solution. The study provides significant insights to readers in the relevant field, including (1) the matrix (GNR) used for doping demonstrates limited ORR electrocatalytic performance, and (2) the soft doping procedure employed does not substantially alter the matrix. Consequently, this enables a thorough investigation of the doping effects on ORR activity and selectivity in various electrolyte solutions.

## 2. Experimental Part

### 2.1. Reagents and Instruments

The chemical compounds used for the experiments were as follows: phosphorus pentoxide (P_2_O_5_, 99%), potassium persulfate (K_2_S_2_O_8_, 99% purity), hydrogen peroxide (H_2_O_2_, 30%), all were acquired from Vetec (Duque de Caxias, RJ, Brazil). Potassium permanganate (KMnO4, 98%) was acquired from Nuclear (Diadema, SP, Brazil). Potassium sulfate (K_2_SO_4_, 99%) was obtained from Proquimios (Rio de Janeiro, RJ, Brazil). Sodium nitrate (NaNO_3_), hydrochloric acid (HCl), potassium hydroxide (KOH), and sulfuric acid (H_2_SO_4_, 98%) were obtained from Merck (Darmstadt, Germany). Ammonia hydroxide (NH_4_OH) solution (28 wt% in H_2_O), hydrazine sulfate (NH_2_NH_2_·H_2_SO_4_, 99%), and multi-walled carbon nanotubes (≥98% purity, containing 6–8 tube walls, O.D. × I.D. × L 10 nm ± 1 nm × 4.5 nm ± 0.5 nm × ~3–6 μm (TEM), 280–350 m^2^/g (BET)) were purchased from Sigma Aldrich (Saint Louis, MO, USA). Platinum (20 wt% of Pt) on graphitized carbon (Pt(20%)C) was purchased from Sigma Aldrich. The water used for the preparation of all solutions was obtained from the Gehaka reverse osmose equipment, with resistivity above 18 MΩ cm and temperature of 25 °C.

A three-electrode glass electrochemical cell was used for the experiments; the cell was composed of a carbon paper HCP030N (geometric area of 3.5 cm^2^), which was used as a counter electrode, a reversible hydrogen electrode, which was used as reference electrode, and Teflon-embedded glassy carbon (GC) disk/Pt ring rotating electrode, which was used as working electrode (the disk and ring had geometric area of 0.196 cm^2^ and 0.11 cm^2^, respectively, with a collection efficiency of N = 0.26—this information was obtained from the manufacturer—Pine Research Instrumentation).

### 2.2. Electrode Preparation

The RRDE (GC disk/Pt ring) electrode was polished with alumina paste (1 µm) and cleaned by sonication, alternating in ultrapure water (Gehaka, resistivity > 18 MΩ cm), isopropyl alcohol, and 0.1 M HClO_4_ (Tedia, suprapure quality) for 5 min in each solvent. The GC disk was subjected to 12 scanning cycles at a sweep rate of 50 mVs^−1^ in the potential range of 0.05–1.2 V. The Pt ring was also subjected to 300 scanning cycles at 900 mVs^−1^ in the potential range of 0.05–1.2 V. Both cycling experiments were performed in 0.1 M HClO_4_ solution saturated with N_2_ (acquired from White Martins, 4.0 of purity); subsequently, the GC disk/Pt ring was washed with ultrapure water and dried with N_2_ flow. A loading of 150 μg cm^−2^ of undoped or doped GNRs on the GC disk surface was obtained by dripping 30 μL of 1.0 mg mL^−1^ aqueous solution of undoped or doped GNRs on the disk surface, which was then dried at room temperature.

### 2.3. Apparatuses, Measurements, and Material Characterization

To perform hydrodynamic linear potential (HLS) and cyclic voltammetry (CV) scanning analyses, a bipotentiostat AFP2 WaveDriver 20–galvanostat (Pine Research Instrumentation) was used—the equipment was connected to AFMSRCE speed modulated rotator.

A PGSTAT128N potentiostat–galvanostat (Autolab) equipped with the FRA2.X module was used during the electrochemical impedance spectroscopy (EIS) experiments. EIS measurements were performed at an open circuit potential with average values of 0.72, 0.89, and 0.82, while the working electrode was placed in the presence of 0.5 M H_2_SO_4_, 0.1 M K_2_SO_4_, and 0.1 M KOH solutions, respectively, in the frequency range of 10 mHz to 100 kHz, with disturbance potential of 10 mV (rms). The ohmic drop resistance, adjusted from a high-frequency EIS intercept, was used to correct each HLS curve. The measured ohmic drop resistances were on average 5.2, 36.8, and 38.5 Ω in 0.5 M H_2_SO_4_, 0.1 M K_2_SO_4_, and 0.1 M KOH solutions, respectively.

The Fourier transform infrared spectroscopy measurements were carried out using Bomen Fourier transform infrared spectrophotometer with a spectral window of 400 to 4000 cm^−1^. The catalyst samples were produced by grinding the dried catalyst powder (approximately 30 µg) with potassium bromide (approximately 35 mg) in a mortar until a fine and homogeneous powder was obtained. After pressing the powder, a translucent tablet was formed.

The Raman spectra were obtained at room temperature using LabRam HR Evolution micro-Raman spectrometer from Horiba Jobin-Yvon, a solid-state laser operating at 633 nm, a standard grating (600 gr/mm), and EMCCD detector (Synapse EM). To avoid overheating and the occurrence of photochemical phenomena, the samples were excited with low-intensity laser (ca. 2 mW). The laser was focused on the sample using a 100 objective (Olympus, MPlan N). The spectra were collected over the course of 12 s.

Elemental analysis (EA) was performed using a Scientific Flash 2000 CNHS/O Elemental Analyzer Thermo Equipment under cycle operating conditions (run time) of 720 s and oven temperature of 950 °C for CHNS determinations and under the cycle (run time) of 400 s and oven temperature of 1060 °C for O determination.

The catalyst nanostructures were characterized by TEM using JEM 2100F (JEOL) or Philips CM200, both operating at 200 kV. The XPS analyses were performed using the PHI Quantera II surface analysis equipment. The Al Kα line (1486.6 eV) was used as the ionization source, operating at 15 kV and 25 W. After performing the background subtraction, the spectra were deconvoluted using a combination of Lorentzian (30%) and Gaussian Voigt (70%) functions.

Thermogravimetric analyses were conducted using Shimadzu TGA-50 thermogravimetric analyzer, with FID Synthetic Air 5.0 gas flow of 50 mL min^−1^, in temperatures ranging from ambient to 900 °C and a heating rate of 10 °C min^−1^; the samples were placed in alumina ceramic crucibles.

### 2.4. GNRs Synthesis and Syntheses of Different Doped GNRs Samples

The synthesis of graphene nanoribbons (GNRs) derived from the chemical oxidation of multi-walled carbon nanotubes (MWCNTs) was carried out utilizing the method previously described elsewhere [7]. Briefly, MWCNTs were dispersed in concentrated H_2_SO_4_ solution containing K_2_S_2_O_8_ and P_2_O_5_ and then heated (with stirring) at 80 °C for 6 h. The resulting solution was diluted in water at 0 °C, filtered through a 0.22 µm nylon membrane, and finally washed with plenty of high-purity water. This pre-oxidized material was re-oxidized in a concentrated H_2_SO_4_ solution containing NaNO_3_ and KMNO_4_ at 30 °C for 2 h, then diluted in high-purity water, and then H_2_O_2_ (30% solution) was added and again diluted with water. After 24 h, this dispersion was centrifuged and washed with HCl and water (10:90), resulting in graphene oxide nanoribbons (GONRs). The GONRs were immersed in hydrazine sulfate and NH_3_OH solution under vigorous stirring, followed by heating to 95 °C for 2 h. After reaching room temperature, the dispersion was filtered initially with a 5% NH_3_OH solution, and washed well with enough high-purity water, finally producing the GNRs.

The doping of GNRs with N and/or S and/or P was performed using NH_4_OH and/or N_2_H_6_SO_4_ and/or K_2_S_2_O_8_ and/or P_2_O_5_; the doping was carried out in two hydrothermal synthesis steps. The first step was executed as summarized in Table 1. This step involved mixing 0.02 g GNR with defined amounts of the dopant or dopants (NH_4_OH and/or N_2_H_6_SO_4_ and/or K_2_S_2_O_8_ and/or P_2_O_5_) and pouring water in a 30 mL beaker; this was followed by heating and constant stirring for 3 h, and, finally, washing/centrifugation several times.

In the second step of synthesis, the product of step 1 was mixed with 30 mL of water or NH_4_OH, together with the respective amount of dopant or dopants (Table 1), and subjected to constant magnetic stirring until complete dispersion was obtained. The resulting solution was heated in an autoclave system at 150 °C for 12 h. The final products were washed by centrifuging using ultrapure H_2_O and dried at 60 °C for 24 h (Figure S1).

There are other well-known methods for N-doping carbon materials such as melamine, used to N-dope commercial carbon black (Vulcan XC 72R) by pyrolyze at different temperatures [41]; acetonitrile, used as a nitrogen source to modify the Ketjen Black EC-600JD carbon support by pyrolyze at 890 °C [42]; and chelates, based on porphyrins N-doping Vulcan XC-72 by pyrolyze at 850 °C [43]. However, we chose to use our previous N-doping method [44] because we knew that the GNR N-doping would result in a relatively low percentage of N-doping, replacing N sources with S and/or P sources to produce GNR’ S- and/or P-doped.

## 3. Results and Discussion

### 3.1. FT-IR and Raman Study

Initially, Fourier Transform infrared spectroscopy (FT-IR) was used to monitor characteristic vibrations; this was done in order to assess any possible changes in the functional groups present on the graphene surface after the doping process. Figure S2a shows the FT-IR spectra for the different doped and undoped GNRs, K_2_S_2_O_8_, and P_2_O_5_ materials.

In general, FT-IR responses for the different doped and undoped GNR materials show peaks related to OH stretching vibration from water, ring stretching, in-plane C-H bending strongly mixed with C–C vibrations or C-N stretching, ring bending vibration or C-P stretching or C–S stretching, out-of-plane ring bending, and C-S stretching [45,46] (Table S1). However, when GNR is doped with nitrogen (GNRN), the ring stretching peak is displaced to a higher wavenumber simultaneously and the C-N stretching is observed at a higher wavenumber as a broad peak (1203 cm^−1^); the ring bending vibration observed in the GNRN is also slightly displaced to a lower wavenumber and the catalyst also exhibits an out-of-plane ring bending strong peak in comparison with the GNR (Figure S2a and Table S1).

When GNR is doped with sulfur (GNRS), the ring stretching peaks are evidenced by the presence of three strong peaks at 1693, 1637, and 1527 cm^−1^; simultaneously, the C-S stretching is observed as a broad small peak at 1086 and a weak peak at 669 cm^−1^ in comparison with the response obtained for the bare GNR (Figure S2a and Table S1)—evidencing the doping of the GNR matrix with S atoms.

When GNR is doped with phosphorous (GNRP), the ring stretching peaks are evidenced by the presence of three well-defined peaks at 1693, 1637, and 1530 cm^−1^; simultaneously, the C-P stretching is observed as a broad strong peak at 1038 cm^−1^ in comparison with the response obtained for the bare GNR (Figure S2a and Table S1).

The ring stretching peaks for GNR doped with nitrogen and sulfur (GNRNS), nitrogen and phosphorous (GNRNP), sulfur and phosphorous (GNRSP), and nitrogen, sulfur, and phosphorous (GNRNSP) can be found to be similarly identical (defined peaks at around 1693, 1640, and 1525 cm^−1^); simultaneously, the C-S stretching is observed as a weak peak at 669 cm^−1^ for those GNR doped with sulfur and the other elements. The GNR doped with nitrogen and the other elements exhibits a broad strong peak at around 1090 cm^−1^, which is most probably related to the stretching of C and the other elements, while the GNR doped with sulfur and phosphorous (GNRSP) exhibits a broad weak peak at around 1068 cm^−1^, which is most probably related to the stretching of C and the other elements (Figure S2a and Table S1). These results help to confirm the doping of GNRs with their respective doping elements.

Figure S2b shows the Raman spectra obtained for GNRs and the doped GNR samples. Clearly, one can observe the intense first-order D band (disorder band) at ≅1328 cm^−1^ and the G band (graphite band) at ≅1597 cm^−1^ [5,47,48,49,50] (Table S2). The higher intensity of the D band in comparison with the G band (I_D_/I_G_ ratio, Table S2) is attributed to the contribution of the bands as defects [48]. Also, the double-resonant signals assigned to 2D [51], D + D′ [51], and 2D′ bands at ≅2651, 2914, and 3214 cm^−1^, respectively, are found to be visible though with lower intensity [48,52,53,54] (Table S2). The D + D′ and 2D′ bands observed in the Raman spectra are related to disorder-induced and second-order Raman overtone of damaged graphene [55]. The displacement of these bands to higher wavelengths suggests a higher degree of disorder in the doped GNRs in comparison with undoped GNRs (as shown in Table S2). Some GNR multilayers present in the samples lead to the emergence of a discrete peak at ≅2468 cm^−1^ (D + D″ band, Table S2), which is typically characteristic of graphene [48]. The displacements observed in the band positions (Table S2) are indicative of the doping of the GNR structure with different elements [29]. Also, the change in the I_D_/I_G_ ratio helps confirm the doping of GNR structures with different elements and the variation in defects [31,34,49,56]. In comparison with undoped GNR (I_D_/I_G_ ratio = 1.72), when N is introduced as a dopant in GNR, the number of defects decreases (resulting in a lower I_D_/I_G_ ratio). Conversely, S doping increases the number of defects yielding a higher I_D_/I_G_ ratio, while P doping changes moderately, as shown in decreases in the defects’ number (resulting in a closer I_D_/I_G_ ratio). Co-doping GNR with NS and NSP discreetly increases the defects’ number (I_D_/I_G_ ratio = 1.79 and 1.76, respectively) and the NP co-doping of GNR is identical in defects’ number (I_D_/I_G_ ratio = 1.72). Finally, SP co-doping of GNR reduces the defects’ number (I_D_/I_G_ ratio = 1.58, as shown in Table S2). The tendency reason in the I_D_/I_G_ ratio for the different materials can be explained by the significant reduction in the number of O-functional groups in GNR doped with nitrogen, leading to a decrease in edges/defects after nitrogen doping (detailed in the next section). On the other hand, in the case of doping with S and/or P, these larger-sized elements (higher than carbon) contribute to an increase in edges/defects after doping.

### 3.2. XPS, EA, and TGA Study

The survey XPS spectra (Figure S3) show the presence of C 1s and O 1s peaks at 285 and 533 eV, respectively, and a very small O KLL peak at 974 eV for all the samples; also, the spectra show the presence of a N 1s peak at 400 eV (Table S3) for the N-containing modified GNRs.

The average values of the mass percentage composition obtained for the modified GNRs are as follows: 87.4 and 10.1% for C 1s and O 1s, respectively, and 4.3% for N 1s (Table S3). These values are quite close to the values corresponding to C and O obtained from the elemental analyses (EA)—where we recorded average values of mass percentage composition of 83.6 and 8.1%, respectively, and 2.3% for N (Table S4). Individually, some of these compositions are even closer to each other (compare Tables S3 and S4).

The results obtained from the EA (Table S4) show that the amount of N is increased considerably for the GNRs doped with N (3.0–6.2 wt.%) in comparison with the undoped GNRs (1 wt.%); the amount of O is significantly diminished in the GNRs doped with nitrogen (lower amount of O:4–5 wt.%) compared to the undoped GNRs (9.7 wt.%); the amount of H is 1.5% on average for the doped GNRs or undoped GNRs; and finally, an amount of 4 wt.% is recorded on average for other elements in the doped GNRs or undoped GNRs. Taking into account the due proportion of the materials, the different catalysts investigated exhibited similar behavior in terms of mass percentage composition of C, O, and N—as shown in Table S3. In summary, the amount of N in doped GNRs varied between 3.0 and 6.2 wt.% (EA results). We are not able to quantify the amount of S and/or P in doped GNRs using the techniques addressed in the present work.

The results obtained from the thermogravimetric analyses showed that the undoped GNRs exhibited mass loss similar to that previously reported in the literature [57,58,59]. In addition, when considering the temperature region—between 552 and 666 °C—within which effective burning of the undoped GNRs occurred, the doped GNRs exhibited variations in the mass loss curve region, ranging from 53 °C (lower) to 23 °C (higher) compared to the mass loss region of the undoped GNRs; furthermore, we noted some residual mass % after 700 °C (Figure S4), which is in agreement with the residual wt.% detected through EA (Table S4). 

**Figure 1:**
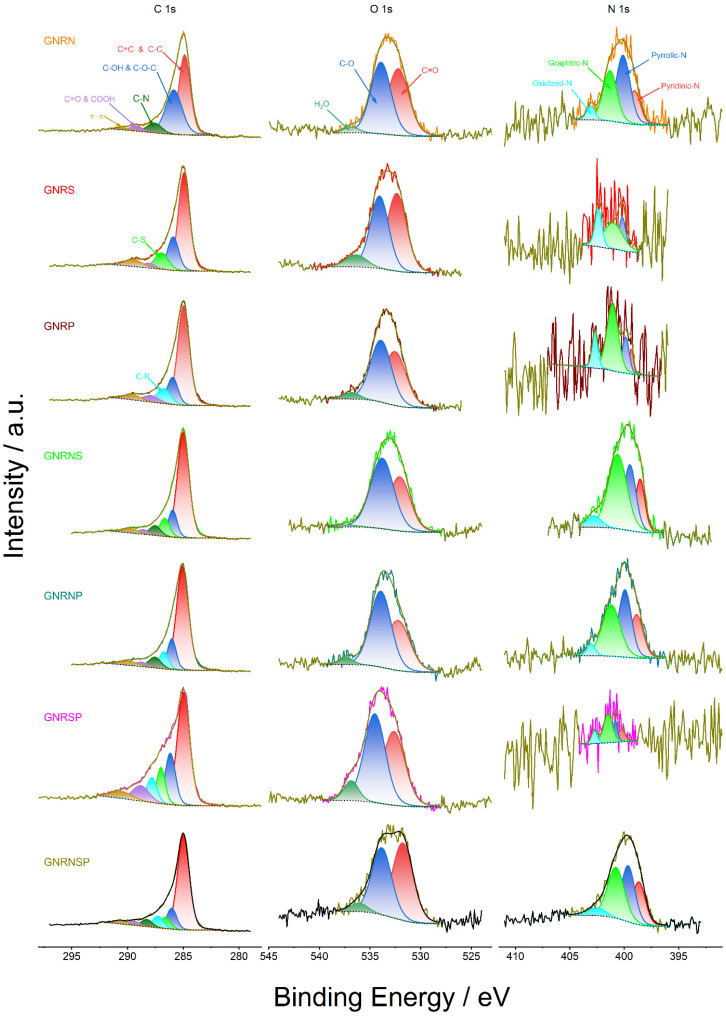
HR-XPS curves for the undoped and doped GNR samples.

Figure 1 shows the C, O, and N 1s core-level XPS spectra obtained for the doped GNR samples after peak deconvolution.

The C 1s spectra show a very accentuated peak and a small shoulder (Figure 1, first column), which were deconvoluted into five to seven peaks attributed to the chemical states C=C & C-C, C–OH & C–O–C, C–S, C–P, C–N, C=O & COOH, and π–π positioned on average at 285, 286, 286.8, 287.1, 287.8, 288.7, and 290.1 eV, respectively (Table S5). Overall, these results are in agreement with the results reported in the literature [11,12,13,44,59,60,61]. The C 1s spectra deconvoluted into the peaks related to the chemical states of C–S and/or C–P and/or C–N for the different catalysts further confirm the modifications produced by these atoms in the GNR structure. Also, on average, the main contributions in terms of % content come from C=C & C-C and C–OH & C–O–C chemical states with 76.7%, followed by (on average) C–S (9.6%), C–P (8.7%), and C–N (6%) (Table S5). The last information confirms the doping of GNRs with S and/or P and/or N.

The O 1s spectra, in general, exhibited a broad peak (Figure 1, second column), which was deconvoluted into three peaks attributed to the chemical states of C=O, C–O, and H_2_O, and positioned on average at 532.3, 534, and 536.6 eV, respectively [11,12,13,44,59,60,61] (Table S5). The main contribution in terms of % content came from the chemical states of C=O and C–O, with an average of 96.5% (Table S5).

The N 1s spectra also displayed a broad peak (Figure 1, third column), which was deconvoluted into the pyridinic-N, pyrrolic-N, graphitic-N, and oxidized-N peaks, positioned on average at 399.1, 400.0, 401.1, and 402.9 eV, respectively [11,12,13,44,59,60,61] (Table S5); here, the main contributions in terms of % content came from pyrrolic-N and graphitic-N chemical states with an average of 28.6 and 44.6%, respectively, followed by pyridinic-N with an average of 15.9% (Table S5).

Figure S5 shows the HR-XPS spectra for P 2p, S 2p, Cl 2p, Mn 2p, and Fe 2p, which are constituted mostly of noise; this shows that the P and S elements exhibited a low signal, and low amounts of contaminants (Cl, Mn, and Fe) remained after the synthesis of undoped GNRs and doped GNRs.

### 3.3. XRD, TEM, and EDX Study

The X-ray diffraction (XRD) technique was used to investigate the effect of the doping process on the crystalline structure of GNRs. Figure S6 shows the diffraction patterns obtained for the undoped and doped GNRs.

The results obtained from the XRD analyses show that the doped GNR exhibits a prominent peak at 2*θ* = 25.8° (0.35 nm), which is typically characteristic of the crystalline peak for the theoretical C graphite with (110) plane (PDF89-8489) and a small peak displacement at 2*θ*, depending on the doping element in comparison with the undoped GNR (Figure S6). Also, there is a small peak at 2*θ* = 42.7–43° (0.21 nm) related to the plane (201) (PDF 89-8489) and a shoulder at 2*θ* = 18.8°, which is typically associated with the doped GNR. These results also help to confirm the doping of GNRs.

**Figure 2:**
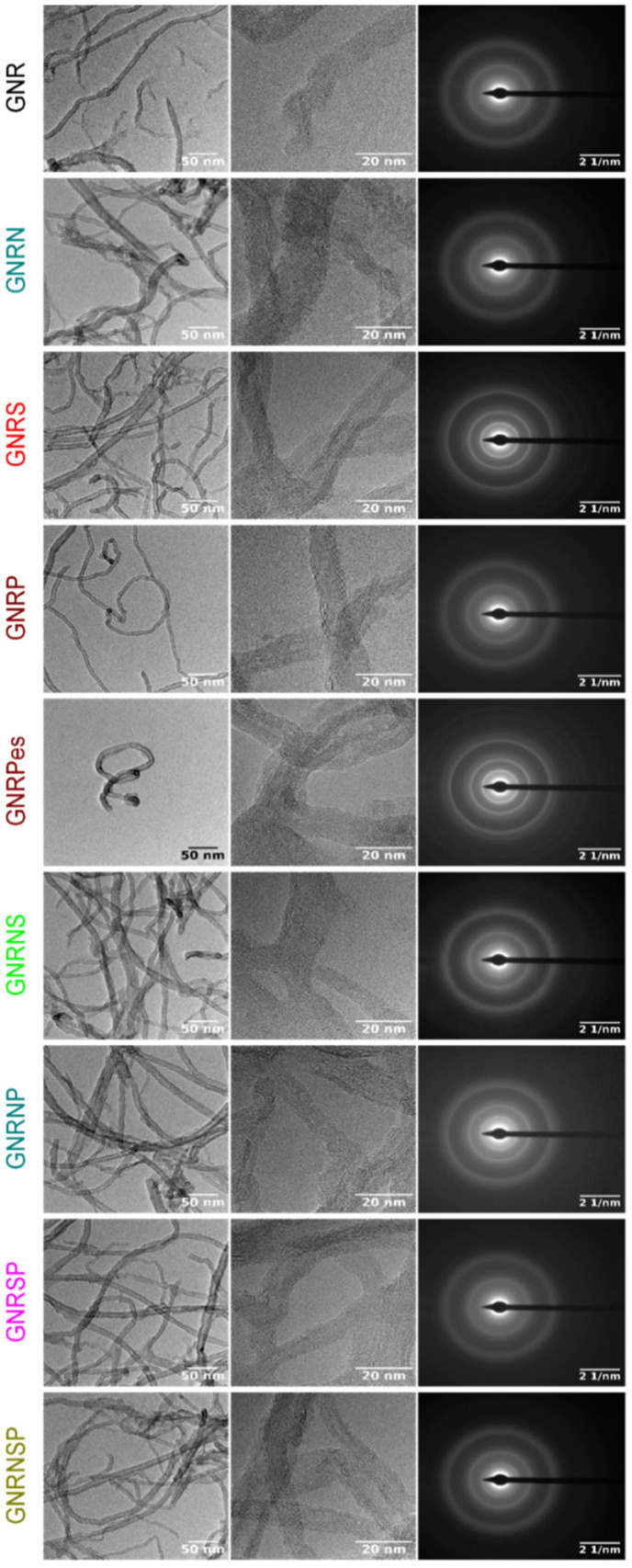
TEM and electron diffraction pattern images for the undoped and doped GNR samples.

From the TEM images (Figure 2 and Figure S7, one can clearly see the GNRs (MWCNT opened structures) with plenty of edges in the structure; however, one is unable to clearly see the differences between the doped and undoped GNR samples. The selected area electron diffraction (SAED) patterns of the samples discreetly display the undoped and doped GNR spots with low crystallinity. Furthermore, the observed ring pattern for all the samples is related to the lack of crystallographic orientation between the GNR samples [62]. For the different doped GNR samples, the EDX mapping images (Figures S8–S10) show a regular distribution of the doping elements (N, S, and P) and a high dispersion of oxygen atoms.

### 3.4. Electrochemical Study—ECSA and CV Profile

**Figure 3:**
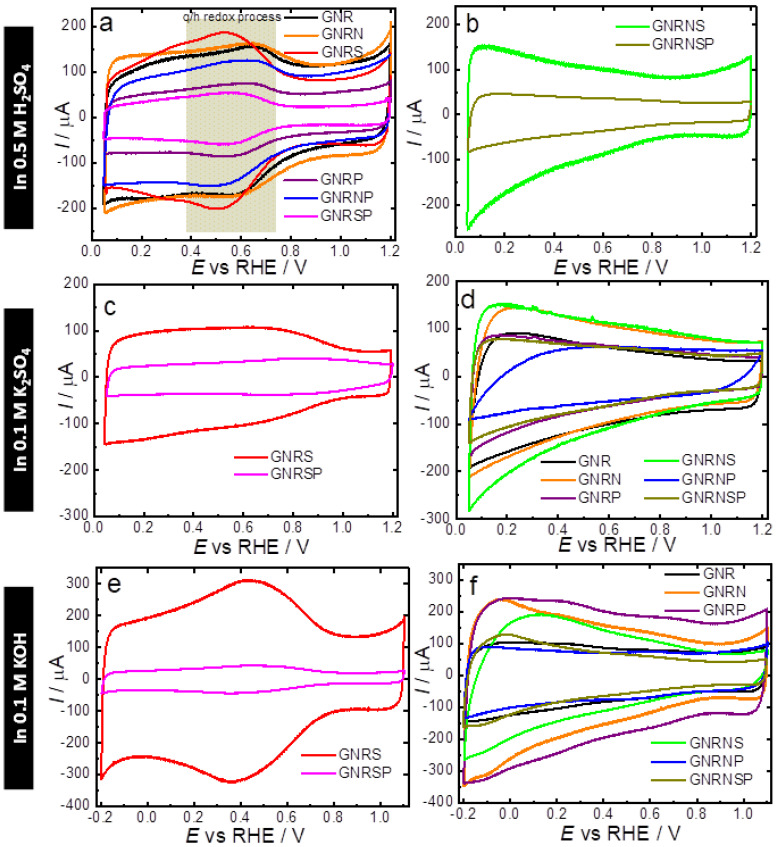
CV obtained for the GC electrodes modified with 150 µg cm^−2^ of undoped or doped GNR applied in N_2_-saturated 0.5 M H_2_SO_4_, 0.1 M K_2_SO_4_, and 0.1 M KOH. Potential scan rate: 50 mV s^−1^ (scans were initiated at 1.2 V).

The undoped and doped GNR samples were characterized electrochemically by cyclic voltammetry (CV) using N_2_-saturated 0.5 M H_2_SO_4_, 0.1 M K_2_SO_4_, and 0.1 M KOH electrolyte solutions; the results obtained are shown in Figure 3a–f. Based on the CV obtained from the N_2_-saturated 0.5 M H_2_SO_4_, the undoped GNR (Figure 3a) exhibited capacitive behavior, characterized by double-layer charging and discharging currents, and redox peaks around 0.6 V, which are typically attributed to the presence of quinone groups on the GNR surface [11,58]. Quinones are known to have high selectivity for the production of H_2_O_2_ when present on the edges and basal planes of carbon nanostructures [63]. During the ORR, these quinone groups can also act as electron acceptors, facilitating efficient electron transfer from the electrode to the reaction intermediates. In general, heteroatom-doped or co-doped GNR samples exhibited two distinguishable patterns of electrochemical behavior (CV shapes) and differences in capacitive current values. Although doping, in general, made the redox pair at ca. 0.6 V less pronounced (GNRP and GNRSP samples, Figure 3a), a closer look at the results showed that the GNRS samples exhibited a more pronounced redox couple related to the quinone/hydroquinone process (q/h); this implies the presence of higher contents of oxygen-functional groups in comparison to other undoped and doped GNR samples—this observation is in good agreement with the XPS results (c.f. Table S3). The noticeable appearance of the q/h redox peak leads to a more symmetrical CV profile with the shape of a pseudo capacitor (c.f. Figure 3) [64,65,66]. On the other hand, an intensification of the smoothing of the q/h peak leads to the formation of a profile that resembles an irregular quadrilateral object (c.f. Figure 3b, GNRNS and GNRNSP samples), which is found to be typically associated with a purer electrical double-layer (DL) capacitor; this capacitor is able to store charge electrostatically through the DL without the contribution of the faradaic q/h process [64,65,66].

Based on the CV obtained in a neutral medium (Figure 3c,d), we observed, in general, that all the undoped or heteroatom-doped or co-doped GNR samples exhibited similar electrochemical behavior with slight changes in the capacitive current values. Regarding the shape of the CV, in neutral media, the GNRS and GNRSP samples (Figure 3c) exhibited relatively less pronounced q/h redox peaks (purer electrical DL capacitor behavior); this implies that the depletion of H^+^ exerts a significant influence over the capacitive ability. 

In the alkaline medium (Figure 3e,f), in general, the CVs exhibited similar shapes for the undoped or heteroatom doped or co-doped GNR and slightly high capacitive currents in comparison with the CVs obtained in the neutral medium; this points to the effect of the doping process on the capacitive ability of graphene nanoribbons. Regarding the q/h redox peaks, the GNRS and GNRSP (Figure 3e) exhibited the most accentuated q/h peaks compared to the other samples (Figure 3f); this shows that the insertion of S in isolation or in combination with P exerts a strong influence over the q/h redox process of the GNR sample, and this leads to a more pseudo capacitive behavior in alkaline media. In general, all the other heteroatom-doped or co-doped GNR samples exhibited similar electrochemical behavior with purer electrical DL capacitor CV profiles and high capacitive current values—of around 350 µA.

Table 2 shows the DL capacitance (C_dl_) and the electrochemical surface area (*ECSA*) values obtained for the undoped and doped GNR samples in different media. Overall, the results obtained show that the C_dl_ and ECSA are highest in acidic media, lower in neutral media, and lowest in alkaline media. The GNRN catalyst recorded the highest *ECSA* in acidic media—ca. 159.4 cm². This increase in *ECSA* can be attributed to two key factors. Firstly, the redox processes associated with the presence of more basic functional groups, such as quinones, pyridinic-N, and pyrrolic-N groups (which require protons for the overall redox processes), significantly contribute to the enhanced capacitance and *ECSA*. Secondly, the introduction of N atoms in the GNR leads to a reduction in the stacking effect on graphene nanoribbons, resulting in a modified electronic structure. This reduction enhances accessibility to the carbon structure, leading to a more pseudo-capacitive behavior and ultimately a higher *ECSA* value [67]. On the other hand, the GNRSP catalyst recorded the lowest *ECSA* in neutral and alkaline media, with values of approximately 37.4 and 8 cm^2^, respectively. These results suggest that the presence of two or three dopants tends to decrease the *ECSA*. The differences in C_dl_ and *ECSA* observed from the use of different electrolyte solutions are related to the differences in the physical–chemical properties of the electrolytes—such as pH, size, and charge of ions in the electrolyte solution (i.e., hydroxyl has a smaller charge-to-mass ratio than sulfate ions), and resistance (c.f. Table S6 and Figure S11), as previously reported in the literature [68]. Particularly GNRP, which exhibited an opposite trend with higher capacitance in an alkaline medium, we attribute this difference to the lower electronegativity of phosphorus atoms compared to carbon atoms and the ability of phosphorus to modulate the charge densities of carbon, allowing negatively charged species to approach the electrode surface and participate in the redox processes. Our results suggest that P-doped samples do not necessarily depend on the presence of protons to exhibit high values of C_dl_ and *ECSA*. However, it is important to note that the behavior of different samples can be influenced by various factors, and further investigation is needed to fully understand and explain the observed variations.

### 3.5. Analysis of ORR Activity and Selectivity

#### 3.5.1. 0.5 M H_2_SO_4_ Solution

**Figure 4:**
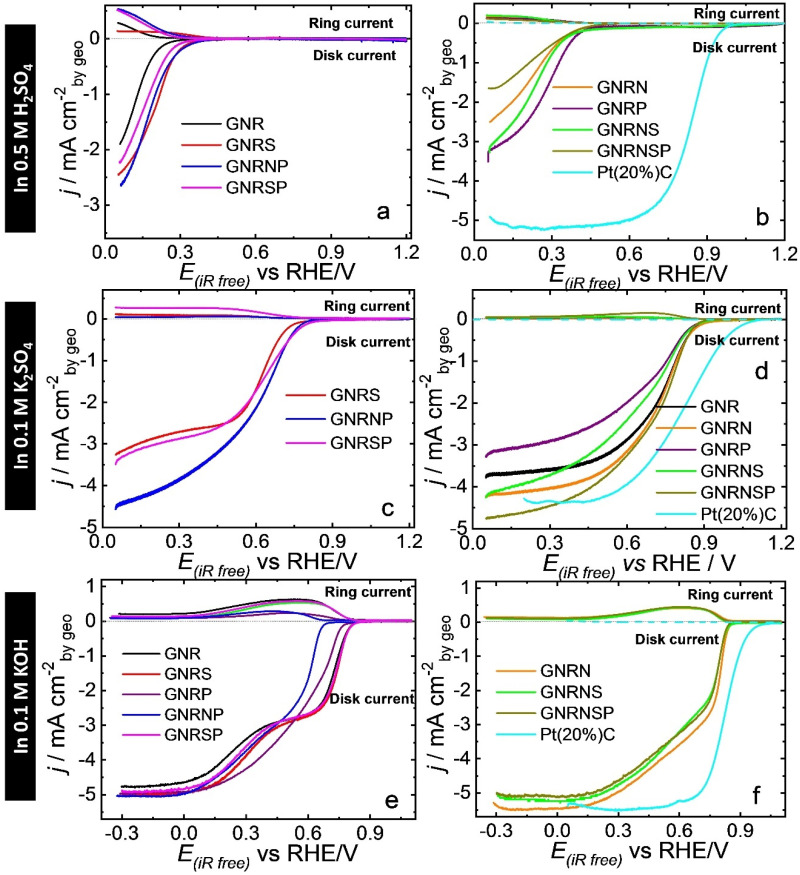
Results obtained from the LSV analysis conducted in a RRDE system for GC modified with 150 µg cm^−2^ of undoped or doped GNR catalysts under the following conditions: scanning at the potential of 10 mV s^−1^; RRDE rotation at 1600 rpm; electrolyte solutions applied: O_2_^−^ saturated 0.5 M H_2_SO_4_, 0.1 M K_2_SO_4_, and 0.1 M KOH (scans were initiated at 0.05 or −0.3 V).

Initially, we evaluated the ORR activity and selectivity of the undoped and doped GNR samples in O_2_-saturated 0.5 M H_2_SO_4_ using linear sweep voltammetry (LSV) in an RRDE system. The results, presented in Figure 4a,b, show that the undoped GNR catalyst had an ORR onset potential of 0.26 V; this shows that the catalyst exhibited relatively less efficient activity compared to metal-based catalysts like Pt [70], Pt alloy encapsulated by nitrogen-doped graphene nanosheets [71], Pt nanoalloy integrated into a hierarchical nitrogen-doped carbon nanotubes (NCNTs) [72], and carbon-based Pt catalysts [73]. After the doping process, the GNR doped with P exhibited the highest improvement in ORR activity among the samples, with an onset potential (E_onset_) of 0.45 V, followed by GNRNS and GNRN (also GNRNSP) with E_onset_ of 0.42 V and 0.41 V, respectively—values obtained for all the samples can be found in Table 2. In terms of selectivity (Figures S16 and S17), the GNRSP and GNRNP catalysts were found to be more favorable to ORR via the 2e^−^ pathway, exhibiting over 90% selectivity (n ≈ 2.2) toward H_2_O_2_ production (X H_2_O_2_), while pristine GNRs recorded 77% selectivity (n ≈ 2.5). On the other hand, the GNRP, GNRNS, and GNRNSP catalysts recorded X H_2_O_2_ of ca. 42% (n ≈ 3.2) on average; this shows that the doping of GNRs with P concomitantly or not with N and S boosted the catalytic activity and favored the occurrence of ORR through the 4e^−^ pathway in acidic media (c.f. Table 2). Furthermore, the accelerated stress test conducted in the potential range of 0.6 to 1.0 V at 1 V s^−1^ (Figure S18) showed that the catalytic performance of the GNRP remained stable even after 10,000 potential cycles; this outcome is confirmed by the undetectable changes in the TEM and electron diffraction pattern images of the catalyst (Figure 2), as well as its scanning TEM (STEM) and EDX mapping images (Figures S8–S10)—named GNRPes (GNRP after electrochemical stability). When comparing the results of our catalysts in an acidic medium to those obtained from the state-of-the-art catalyst, it is evident that the Pt(20%)C catalyst exhibits higher electroactivity toward ORR. Specifically, it displays an E_onset_ value of 0.99 V, an E_1/2_ value of 0.84 V, and a limit current density of −5.17 mA cm^−2^ (c.f. Figure 4). Additionally, it shows a preference for H_2_O production, with an X HO_2_^−^ value of 3.4% (Figure S16), and a corresponding n_av_ of 3.9 (Figure S17).

#### 3.5.2. 0.1 M K_2_SO_4_ Solution

In a neutral medium (Figure 4c,d), the doping process only led to improvements in ORR catalytic activity when the GNR was doped with nitrogen. The undoped GNR catalyst exhibited an ORR onset potential of 0.89 V, while the GNRN catalyst exhibited a modest improvement with E_onset_ of 0.90 V. The other heteroatom-doped and co-doped catalysts exhibited E_onset_ lower than 0.89 V—see the values in Table 2. 

When the GNR was doped with S, for instance, the E_onset_ decreased significantly from 0.89 to 0.76 V; this outcome shows that the insertion of S atoms in the GNR provoked a negative impact on the catalytic activity, and the decrease in O content reflected the observed catalytic deactivation. In terms of selectivity (Figures S16 and S17), the GNR doped with phosphorus and sulfur favored the occurrence of ORR via the 2e^−^ pathway; for illustration purposes, GNRSP exhibited 53% selectivity toward H_2_O_2_ production (n ≈ 2.9), while pristine GNR exhibited 5.2% selectivity (n ≈ 3.9). On the other hand, GNRs doped with nitrogen (GNRN, GNRNP, GNRNS, and GNRNSP samples with X HO_2_^−^ ≈ 12% and n ≈ 3.8, on average) favored the occurrence of ORR via the 4e^−^ pathway. Just as we observed in the acidic medium, the Pt(20%)C catalyst demonstrates enhanced electroactivity toward ORR even in a neutral electrolyte solution. This is evidenced by its E_onset_ = 1.1 V, E_1/2_ = 0.82 V, and limit current density = −4.36 mA cm^−2^ (Figure 4). In addition, it is more selective to H_2_O production (X HO_2_^−^ = 0.03%, Figure S16) with n_av_ = 4.0 (Figure S17).

#### 3.5.3. 0.1 M KOH Solution

In general, the LSV curves in an alkaline medium (Figure 4e,f) show the characteristic behavior of a 2e^−^ (from around 0.8 to 0.4 V) + 2e^−^ (from around 0.2 to −0.3 V) mechanism, in accordance with the behavior of X HO_2_^−^ (Figure S16) and n_av_ (Figure S17). 

The doping of GNRs with nitrogen caused a significant effect on the catalytic performance in ORR. The GNRN catalyst exhibited an ORR onset potential of 0.86 V, while the undoped GNR catalyst recorded an onset potential of 0.83 V. Also, our GNRN catalyst exhibits comparable behavior to that presented in Figure 9a,b by Liu et al. [74]. Both the GNRNSP and GNRNS catalysts recorded E_onset_ of 0.85 V; this result points to an improvement in catalytic performance following the insertion of N, S, and P groups concomitantly into the GNR matrix. Compared to the GNRs, no improvements were observed in the GNRS and GNRSP samples; however, when these catalysts were doped with P (GNRP and GNRNP catalysts), they recorded E_onset_ values lower than 0.83 V; this shows that P-doping, either alone or in combination with other elements, leads to the loss of catalytic activity for graphene matrix in alkaline media. The MWCNT oxidized and doped with N and S (MWCNToxNS) produced by Wierzbicki et al. [75] results in better E_1/2_ value (better activity and selectivity to H_2_O) in comparison with our catalysts. In terms of selectivity, the undoped GNR catalyst exhibited the highest selectivity toward H_2_O_2_ production, reaching ca. 56% (n ≈ 2.9) in the potential range of −0.3 to 0.7 V. In line with previous studies reported in the literature [11,44], GNRs presented two distinct potential regions with high selectivity. The first region, between 0.70 and 0.35 V, favored H_2_O_2_ formation (X HO_2_^−^ = 90%, n ≈ 2.2), while the second region, between 0.30 and −0.30 V, favored water formation (X HO_2_^−^ = 30%, n ≈ 3.2). It should be noted, however, that heteroatom doping or co-doping of GNRs is found to decrease H_2_O_2_ selectivity, particularly when GNRs are doped with P and N—this results in X HO_2_^−^ lower than 30% (n ≥ 3.4). When comparing the results of our catalysts to Pt-based catalyst in an alkaline medium, as expected, Pt(20%)C is more electroactive toward ORR presenting E_onset_ = 1.02 V, E_1/2_ = 0.82, and limit current density = −5.51 mA cm^−2^ (Figure 4). In addition, it is more selective to H_2_O production (X HO_2_^−^ = 3.4%, Figure S16) with n_av_ = 3.9 (Figure S17).

### 3.6. Comparative Analyses and Assessment of Trends

The results obtained from the characterization of the samples investigated in this study showed that, in general, the heteroatom doping or co-doping of GNRs did not lead to significant structural modifications (see TEM results). However, the analysis of the surface chemistry of the materials helped identify the presence of a doping process, which led to the insertion of the heteroatom concomitantly with oxygen depletion, as well as variation in defects (with no present clear influence on the ORR activity and selectivity toward H_2_O_2_, as pointed out below).

In addition, the results also showed that the electrolyte solution [76] and heteroatom doping or co-doping [77,78,79,80] exerted considerable influence on ORR activity and selectivity in the doped GNR samples (including in comparison with the undoped GNR). Among the dopant elements investigated, N (mainly in the form of Pyrrolic-N and Graphitic-N) was found to be the most easily inserted (and detected) in the carbon matrix.

The results obtained from the electrochemical analysis showed that heteroatom insertion and oxygen depletion after doping also affected the visual intensity of the q/h redox peak and the voltammetric profile, and this exerted influence over the C_dl_ and ECSA values. Heteroatom-doped or co-doped GNR samples exhibited two distinct patterns of electrochemical behavior and differences in capacitive currents. The doped samples that exhibited a more noticeable q/h redox couple, with higher contents of O-groups, had a more symmetrical CV profile, which resembled that of a pseudo capacitor; overall, these samples exhibited lower ORR activity with higher selectivity toward H_2_O_2_ formation in both acidic, neutral, and alkaline media.

On the other hand, samples with CV profiles that indicated the absence or smoothing of the q/h peak exhibited a profile that resembled a pure electrical double-layer capacitor; overall, these samples exhibited enhanced ORR activity and higher selectivity toward water (or OH^−^) formation—this is in line with the catalyst responses shown in other works previously reported in the literature [24,26,29,30,31,33]. Essentially, these results also suggest that the noticeable appearance of the redox couple peaks related to the q/h process in an electrochemical profile is a strong indicator of the selective formation of H_2_O_2_, and this observation can be applied to ORR in different media.

In general, N-doping yielded the most efficient result in terms of ORR-4e^−^-catalytic performance for solutions with high pH. For illustration purposes, N and NSP-doping (GNRN and GNRNSP samples) improved ORR catalytic activity in a neutral medium. These findings regarding the catalysts doped and co-doped with N can be explained in accordance with the Xia et al. [19] model. However, in summary, we assume that catalysts doped and co-doped mainly with graphitic-N and pyridinic-N species (around 60% in total of both species) are favorable to OH^−^ production [44]. The nitrogen atoms with lone pairs of electrons, and with a strong affinity for electrons, produce changes in the charge distribution, electronic state, and spin densities of carbon atoms (sp^2^-hybridized carbon skeleton), promoting a considerable density of positive charges able to act as active sites for oxygen reactions [81,82]. The protonation of N groups introduced during the doping process on the catalyst surface may hinder charge delocalization, thereby compromising the electrocatalytic activity in acidic media and promoting the formation of H_2_O_2_.

On the other hand, the doping of graphene nanoribbons with S and P (GNRS and GNRP, respectively) led to improvements in ORR activity only in acidic media, with the occurrence of ORR seen to be favored under the 4e^−^ pathway. Interestingly, in neutral and alkaline media, the doping of GNRs with S and P led to a decline in ORR activity; this outcome can be attributed to the ability of S and P to generate sulfonic or phosphonic acid groups that can act as proton donors, facilitating the transfer of electrons [83,84]. The results obtained from the experiments conducted in this study also showed that the co-addition of sulfur and phosphorus to graphene nanoribbons (GNRSP) led to a higher formation of H_2_O_2_ in all the three medium conditions evaluated in the study; this is likely because of the adsorption of oxygen molecules in the Pauling-type form in the carbon atoms near the sulfur or phosphorus atoms on the catalyst surface, which made it easier for the HOOH or OOH^−^ to be released after the two-electron transfer in the ORR process [85,86]. Furthermore, our findings also showed that the addition of two or three elements to graphene does not lead to a significant enhancement of its catalytic activity. 

To gain insights into the ORR mechanism in different samples and media, Tafel plots were constructed for selected catalysts, namely, GNRSP and GNRNS, as depicted in Figure S19. Generally, the Tafel slopes of both electrocatalysts are relatively similar, indicating that the introduction of heteroatoms through doping does not significantly alter the reaction pathway. However, notable differences can be observed in their catalytic activities when exposed to alkaline and acidic media. In alkaline conditions, which exhibit an average Tafel slope of 60 mV dec^−1^, a faster and higher catalytic activity is observed. On the other hand, at lower pH values, the slope values are relatively close and higher, averaging around 100 mV dec^−1^. This suggests the involvement of additional reaction steps. The difficulty of the ORR in acidic media may be attributed to the protonation of functional groups within the carbon matrix. This protonation reduces the interaction with molecular oxygen, impairing the ORR and requiring high overpotentials for O_2_ adsorption and subsequent reduction to occur. In contrast, in alkaline media, the deprotonated functional groups exhibit a negative nature, facilitating the removal of OH^−^ ions and promoting the adsorption of O_2_ on the carbon matrix surface. These observations highlight the influence of pH on the ORR process, with acidic media presenting challenges due to functional group protonation, while alkaline media provide a favorable environment for O_2_ adsorption and subsequent reduction.

## 4. Conclusions

In summary, in the present study, we successfully produced carbon catalysts doped and co-doped with N, S, and P using the same graphene (GNR) matrix. A wide-ranging analysis was conducted in order to evaluate the impact of doping on ORR catalytic activity and selectivity in acidic, neutral, and alkaline media. The results obtained from the characterization of the materials investigated showed that the doping process resulted in the insertion of heteroatoms and oxygen depletion, with nitrogen (in the form of pyrrolic-N and graphitic-N) being the most easily inserted among the dopants. Electrochemical characterization analysis showed that the insertion of heteroatoms and oxygen depletion affects the intensity of the q/h redox peak and voltammetric profile, leading to differences in capacitive currents and ORR selectivity. The results obtained from the electrochemical characterization analysis showed that the noticeable intensity of the redox couple peaks related to the quinone/hydroquinone process in a CV profile is a strong indicator of ORR selectivity toward H_2_O_2_ formation, and this observation can be applied for ORR in different media. In terms of the catalytic performance of the elements used for doping GNRs, nitrogen was found to be the most efficient element (its application led to significant improvements in catalytic activity) for solutions with high pH, while sulfur and phosphorus improved ORR activity only in an acidic medium. The co-doping of GNRs with S and P resulted in ORR selectivity toward the formation of hydrogen peroxide in all the three media investigated (acidic, neutral, and alkaline). The results of the study also showed that co-doping with two or three elements did not lead to significant catalytic improvements. The findings of the present study provide useful insights into carbon functionalization and can serve as a pivotal roadmap for the design of more formidable and versatile catalytic materials based on functionalized carbon.

## Figures and Tables

**Table 1:** Summary of the first and second steps of the synthesis.

Catalyst	First Step Involved Heat Treatment at 95 °C for 3 h (Component Amounts as Shown Below), Followed by 10 Cycles of Washing/Centrifugation (7000 rpm) with Water Replacement.						Second Step Was Executed in Autoclave System for 12 h at 150 °C (Component Amounts as Shown Below), Followed by 10 Cycles of Washing/Centrifugation (7000 rpm) with Water Replacement; Subsequently, the Material Was Dried at 60 °C for 24 h			
	GNR (g)	K_2_S_2_O_8_ (g)	P_2_O_5_ (g)	NH_4_OH (mL)	N_2_H_6_SO_4_ (g)	H_2_O (mL)	K_2_S_2_O_8_ (g)	P_2_O_5_ (g)	NH_4_OH (mL)	H_2_O (mL)
GNRN	0.02	-	-	1	1.06	30	-	-	30	-
GNRS	0.02	0.2	-	-	-	30	0.2	-	-	30
GNRP	0.02	-	0.2	-	-	30	-	0.2	-	30
GNRNS	0.02	0.2	-	1	1.06	30	0.2	-	30	-
GNRNP	0.02	-	0.2	1	1.06	30	-	0.2	30	-
GNRSP	0.02	0.2	0.2	-	-	30	0.2	0.2	-	30
GNRNSP	0.02	0.2	0.2	1	1.06	30	0.2	0.2	30	-

**Table 2:** Comparative analysis of the physical–chemical properties and ORR parameters of the undoped and doped GNR catalysts in different media. The table provides the following relevant data for the catalysts investigated: C_dl_ values obtained from the data in Figures S12–S14, ECSA values obtained from the data in Figure S15, ORR onset potential values (E_onset_), potential at half limiting current density values (E_1/2_), number of electrons obtained from the RRDE plot (n_av_), and peroxide percentage (X HO_2_^−^(%)) obtained from the data in Figure 4 and Figures S16 and S17.

Catalyst	Dopant Element (at%) *	C_dl_ (µF)	ECSA ** (cm^2^)	Electrolyte	E_onset_ (V_RHE_) ***	E_1/2_ (V_RHE_)	X HO_2_^−^ (%) ****	n_av_ ****
GNR	-	2248.4	132.3	0.5 M H_2_SO_4_	0.26	0.13	76.7	2.5
GNR	-	1265.8	74.5	0.1 M K_2_SO_4_	0.89	0.73	5.2	3.9
GNR	-	1287.6	58.5	0.1 M KOH	0.83	0.66	55.7	2.9
GNRN	N (2.5 at%)	2709.5	159.4	0.5 M H_2_SO_4_	0.41	0.24	57.7	2.8
GNRN	N (2.5 at%)	1228.4	72.3	0.1 M K_2_SO_4_	0.90	0.72	5.3	3.9
GNRN	N (2.5 at%)	1454.4	66.1	0.1 M KOH	0.86	0.74	29.9	3.4
GNRS	S (nq)	2262.4	133.1	0.5 M H_2_SO_4_	0.33	0.20	52.3	3.0
GNRS	S (nq)	1394.9	82.1	0.1 M K_2_SO_4_	0.76	0.62	23.8	3.5
GNRS	S (nq)	1921.8	87.4	0.1 M KOH	0.83	0.66	41.4	3.2
GNRP	P (nq)	1409.4	82.9	0.5 M H_2_SO_4_	0.45	0.28	35.3	3.3
GNRP	P (nq)	754.5	44.4	0.1 M K_2_SO_4_	0.88	0.68	13.7	3.7
GNRP	P (nq)	2316.8	105.3	0.1 M KOH	0.79	0.58	26.7	3.5
GNRNS	N (3.6 at%) S (nq)	1190.0	70.0	0.5 M H_2_SO_4_	0.42	0.24	41.5	3.2
GNRNS	N (3.6 at%) S (nq)	1112.1	65.4	0.1 M K_2_SO_4_	0.88	0.66	12.5	3.7
GNRNS	N (3.6 at%) S (nq)	892.3	40.6	0.1 M KOH	0.85	0.71	30.5	3.4
GNRNP	N (4.3 at%) P (nq)	2389.6	140.6	0.5 M H_2_SO_4_	0.33	0.18	91.5	2.2
GNRNP	N (4.3 at%) P (nq)	667.5	39.3	0.1 M K_2_SO_4_	0.79	0.60	11.9	3.8
GNRNP	N (4.3 at%) P (nq)	906.9	41.2	0.1 M KOH	0.69	0.53	30.1	3.4
GNRSP	P (nq) S (nq)	722.5	42.5	0.5 M H_2_SO_4_	0.30	0.17	94.5	2.1
GNRSP	P (nq) S (nq)	635.9	37.4	0.1 M K_2_SO_4_	0.80	0.62	53.1	2.9
GNRSP	P (nq) S (nq)	175.1	8.0	0.1 M KOH	0.83	0.65	48.7	3.0
GNRNSP	N (4.6 at%) P (nq) S (nq)	881.4	51.8	0.5 M H_2_SO_4_	0.41	0.25	48.6	3.0
GNRNSP	N (4.6 at%) P (nq) S (nq)	735.5	43.3	0.1 M K_2_SO_4_	0.87	0.71	18.9	3.6
GNRNSP	N (4.6 at%) P (nq) S (nq)	624.7	28.4	0.1 M KOH	0.85	0.72	32.4	3.3

* Value estimated from XPS data (Table S3). nq = detected by XPS, EA, and/or EDX but not quantified. ** The ECSA values were estimated by dividing C_dl_ by the specific capacitance. The specific capacitance values were estimated based on the capacitance of an atomically smooth planar carbon-based material surface per unit area under acidic (17 µF cm^−2^), neutral (we make the same assumption as the acidic medium: 17 µF cm^−2^), and alkaline (22 µF cm^−2^) conditions, as described in ref. [69]. The Δ*I* values were divided by 2 ((Δ*I* = *I*_a_ − *I*_c_)/2), and the current values obtained were measured at the OCP as a function of ν (Figure S15). The slopes of these plots give the C_dl_ values, which are used to determine the ECSA. *** The E_onset_ values were determined based on the point at which the disk current density reached −0.05 mA cm^−2^. ****. The X HO_2_^−^(%) and n_av_ values were calculated (see Equations (S1) and (S2)) within the potential range starting from the lower potential (the potential at which the LSV curve begins) and extending to the onset (E_onset_).

## Data Availability

Research data will be available when requested.

## Supplementary Materials

The following supporting information can be downloaded at https://www.mdpi.com/article/10.3390/nano13212831/s1. Reference [87] is cited in the supplementary materials.
