# Supplementary material for: Exploring the Potential of Heteroatom-Doped Graphene Nanoribbons as a Catalyst for Oxygen Reduction
Source: Nanomaterials (Basel). 2023 Oct 26;13(21):2831. doi: 10.3390/nano13212831 (PMC10650208; doi:10.3390/nano13212831)
Supplement: Supplementary file 1 [file nanomaterials-13-02831-s001.zip › nanomaterials-2570255-supplementary.pdf]

Supporting information for

Exploring the Potential of Heteroatom-Doped Graphene Nanoribbons as a Catalyst for Oxygen Reduction

Eduardo S. F. Cardoso[1], *Guilherme V. Fortunato*[2], *Clauber D. Rodrigues*[3], *Marcos R. V. Lanza*[2],

Gilberto Maia[1,*]

[1]Institute of Chemistry, Federal University of Mato Grosso do Sul, Av. Senador Filinto Muller 1555, Campo Grande, MS 79074-460, Brazil

[2]São Carlos Institute of Chemistry, University of São Paulo, Avenida Trabalhador São-Carlense 400, São Carlos, SP 13566-590, Brazil

[3]State University of Mato Grosso do Sul; Rua Rogério Luis Rodrigues s/n, Gló riade Dourados, MS 79730-000, Brazil

*Corresponding author. Tel: +55 67 3345 3596. E-mail: gilberto.maia@ufms.br (Gilberto Maia)

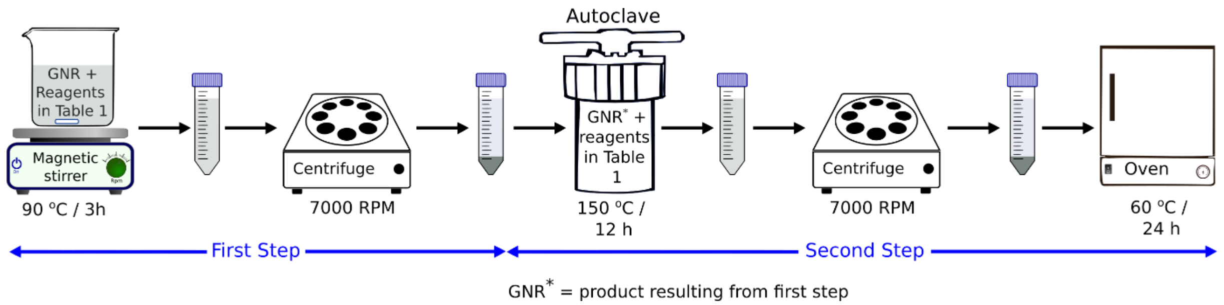

Figure S1. Scheme illustrating the modification of GNR to produce GNRN, GNRS, GNRP, GNRNS, GNRNP, GNRSP, and GNRNSP.

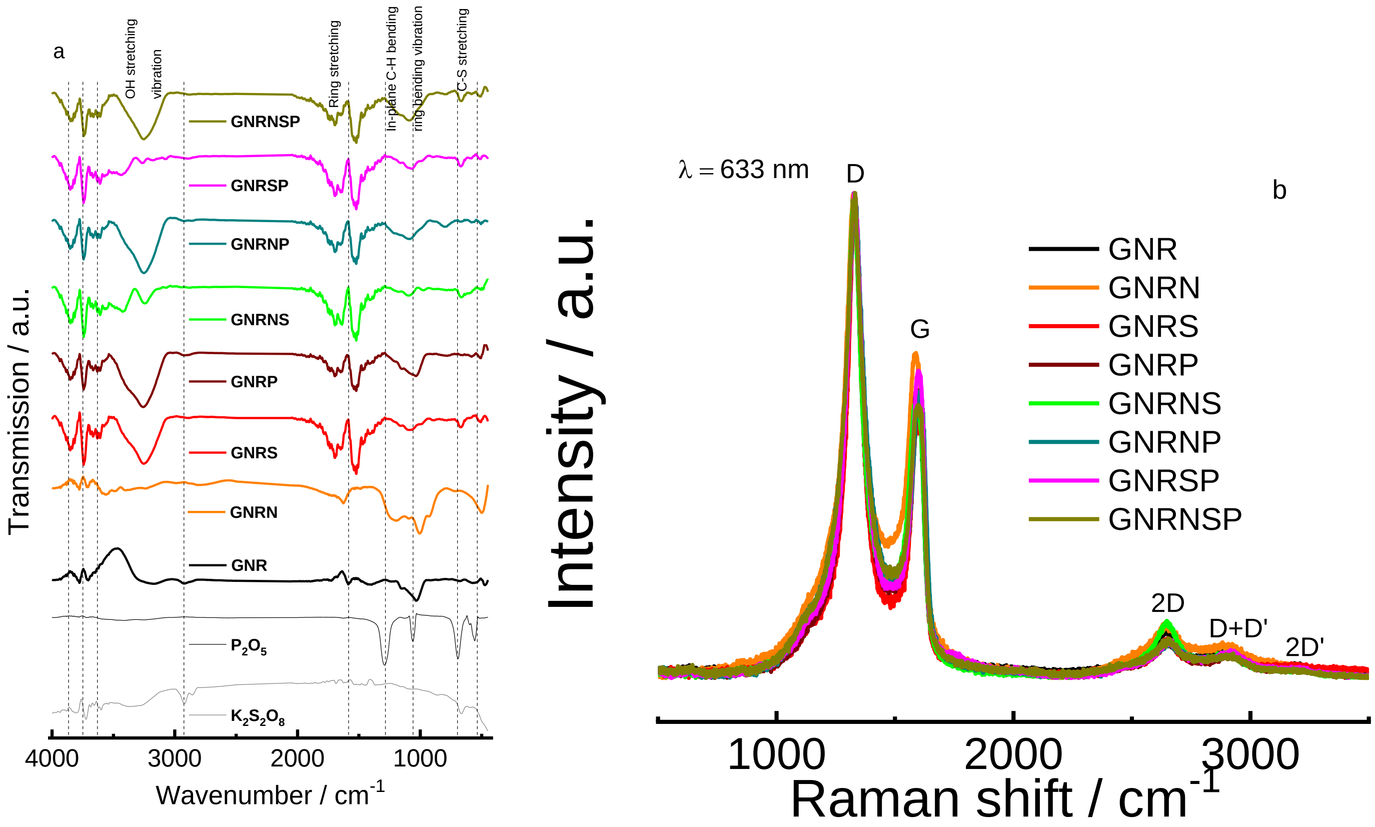

Figure S2. a) FT-IR spectra for different samples of doped and undoped GNR, $K_2S_2O_8$, and P_2O_5. b) Raman spectra for the undoped and doped GNR samples.

Table S1. FT-IR attributions for the different doped and undoped GNR samples.

Catalyst	Attribution [1,2]					
	OH stretching vibration from water (cm^{-1})	Ring stretching (cm^{-1})	In-plane C-H bending strongly mixed with C—C vibrations or C-N stretching (cm^{-1})	Ring bending vibration or C-P stretching or C—S stretching (cm^{-1})	Out-of-plane ring bending (cm^{-1})	C-S stretching
**GNR**	3,182 (discreet broad peak)	1,587 (weak peak)	1,150 (weak peak)	1,026 (broad strong peak)	-	
**GNRN**	-	1,626 (weak peak)	1,203 (broad peak)	1,005 (strong peak)	500 (strong peak)	
**GNRS**	3,254 (broad strong peak)	Strong peaks at 1,693-1,637 (exhibited two peaks) and 1,527	1,086 (broad small peak)		-	669 (weak peak)
**GNRP**	3,256 (broad strong peak)	Well-defined peaks at 1,693-1,637 (exhibited two peaks) and 1,530	1,038 (broad strong peak)		-	-
**GNRNS**	3,240 (weak peak)	Well-defined peaks at 1,693-1,637 (exhibited two peaks) and 1,522	1,095 (broad weak peak)		-	667 (weak peak)
**GNRNP**	3,252 (broad strong peak)	Well-defined peaks at 1,693-1,649 (exhibited two peaks) and 1,528	1,088 (broad strong peak)		-	-
**GNRSP**	-	Well-defined peaks at 1,693-1,637 (exhibited two peaks) and 1,526	1,068 (broad weak peak)		-	669 (weak peak)
**GNRNSP**	3,254 (broad strong peak)	Well-defined peaks at 1,693-1,637 (exhibited two peaks) and 1,522	1,088 (broad strong peak)		-	667 (weak peak)

Table S2. Raman bands attributions for the different doped and undoped GNR catalysts and the I_D/I_G ratio.

Catalyst	Band (cm^{-1})						I_D/I_G
	D	G	D+D''	2D	D+D'	2D'	
**GNR**	1328	1598	2460	2644	2904	3197	1.723
**GNRN**	1324	1587	2474	2646	2904	3210	1.508
**GNRS**	1330	1599	2470	2654	2922	3207	2.001
**GNRP**	1328	1599	2487	2649	2916	3208	1.697
**GNRNS**	1327	1589	2464	2645	2905	3221	1.789
**GNRNP**	1331	1605	2463	2656	2919	3234	1.724
**GNRSP**	1329	1600	2483	2659	2923	3218	1.575
**GNRNSP**	1328	1600	2442	2655	2920	3219	1.757

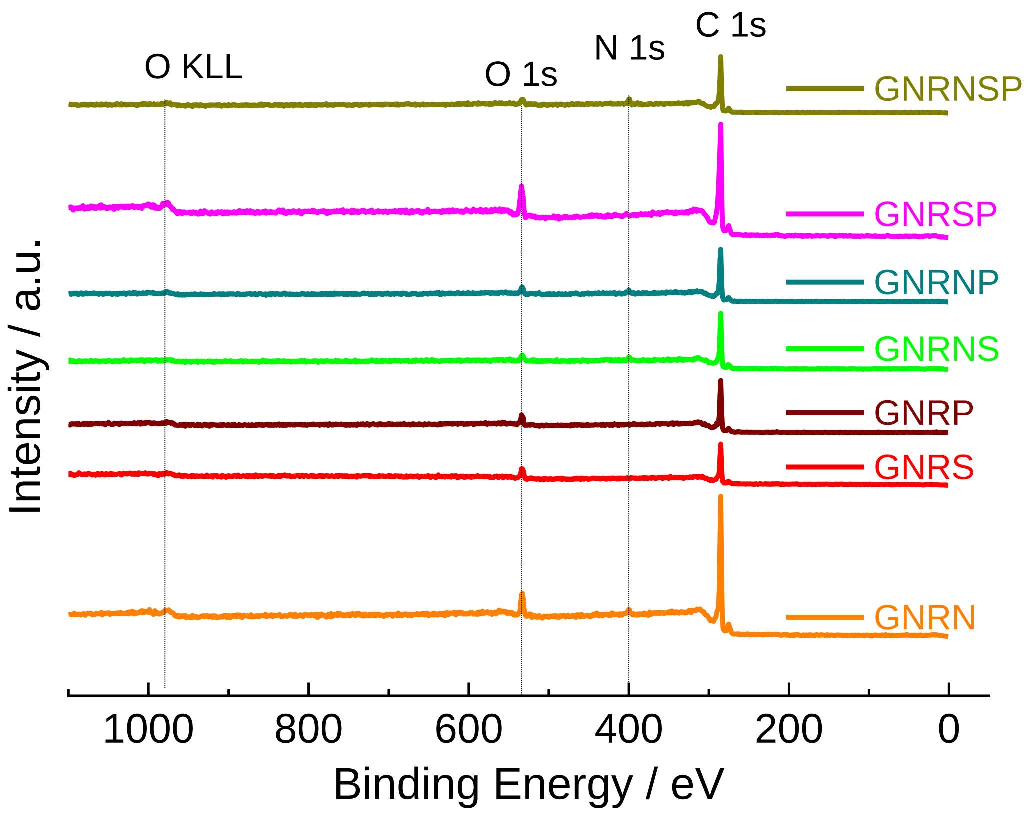

Figure S3. XPS survey spectra of the doped GNR samples.

Table S3. Positions, relative sensitivity factors (R.S.F.), atomic and mass percentages obtained from the survey XPS spectra shown in Figure S2 for the doped GNR samples investigated.

Catalyst	Name	Position (eV)	R.S.F.	Content (at. %)	Content (mass %)
GNRN	C 1s	285.00	1.0	89.5	86.9
	N 1s	400.00	1.8	2.5	2.8
	O 1s	533.00	2.93	8.0	10.3
GNRS	C 1s	285.0	1.0	87.8	84.4
	O 1s	533.0	2.93	12.2	15.6
GNRP	C 1s	285.0	1.0	91.0	88.4
	O 1s	534.0	2.93	9.0	11.6
GNRNS	C 1s	285.0	1.0	91.0	89.0
	N 1s	400.0	1.8	3.6	4.0
	O 1s	533.0	2.93	5.4	7.0
GNRNP	C 1s	285.0	1.0	89.7	87.3
	N 1s	400.0	1.8	4.3	4.9
	O 1s	533.0	2.93	6.0	7.8
GNRSP	C 1s	285.00	1.0	89.7	86.7
	O 1s	534.00	2.93	10.3	13.3
GNRNSP	C 1s	285.0	1.0	91.2	89.3
	N 1s	399.0	1.8	4.6	5.3
	O 1s	533.0	2.93	4.2	5.4

Table S4. Elemental analyses of the undoped and doped GNR samples.

Catalyst	N (wt.%)	C (wt.%)	H (wt.%)	S (wt.%)	O (wt.%)	Others(wt.%)
**GNR**	1.0	85.2	0.9	0.0	9.7	3.2
**GNRN**	3.2	85.7	1.0	0.3	4.0	5.8
**GNRS**	0.2	84.9	1.9	0.0	8.4	4.6
**GNRP**	0.5	84.3	1.4	0.1	8.8	4.9
**GNRNS**	5.9	85.8	1.6	0.0	5.0	1.7
**GNRNP**	3.0	85.2	1.1	0.0	4.9	5.8
**GNRSP**	0.0	85.2	1.8	0.0	9.8	3.2
**GNRNSP**	6.2	84.0	2.5	0.0	4.8	2.5

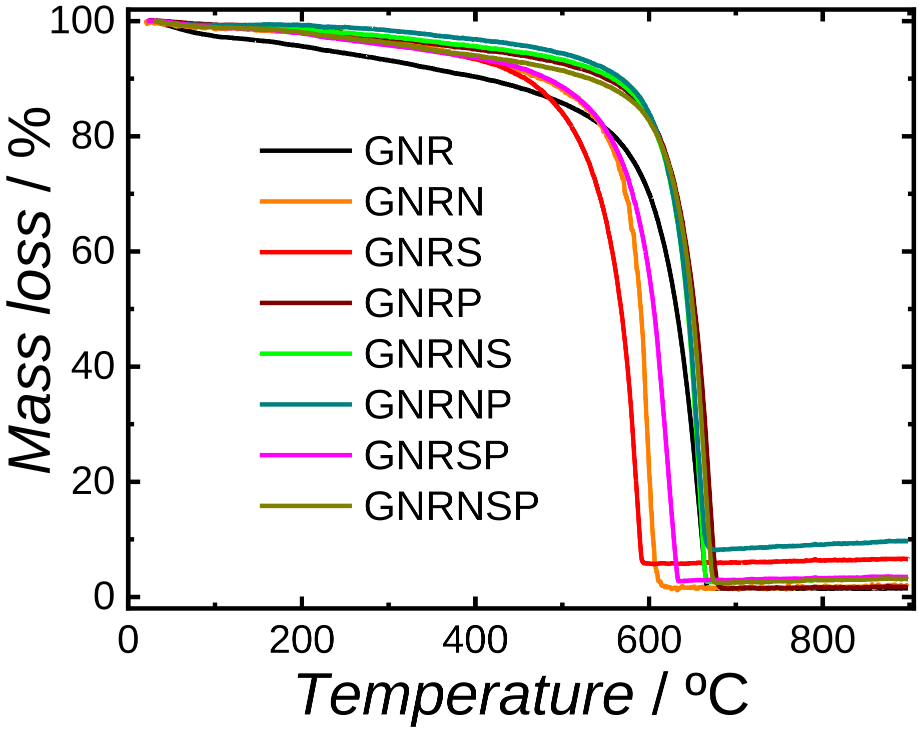

Figure S4. TG curves for the undoped and doped GNR samples.

Table S5. Positions and percentages of the content of functional groups or chemical states present in doped GNR samples obtained from high–resolution XPS spectra shown in Figure 3.

Catalyst	Name	Chemical state	Position (eV)	% content
**GNRN**	O 1s	C=O	532.2	47.9
		C–O	533.9	49.0
		H_2O	536.9	3.1
	C 1s	C=C & C-C	284.9	49.3
		C–OH & C–O–C	285.9	38.9
		C–N	287.6	7.3
		C=O & COOH	289.2	3.2
		π–π	290.3	1.3
	N 1s	Pyridinic-N	399.1	18.2
		Pyrrolic-N	400.1	44.2
		Graphitic-N	401.3	32.4
		Oxidized-N	403.7	5.2
**GNRS**	O 1s	C=O	532.4	48.8
		C–O	534.1	43.2
		H_2O	536.4	8.0
	C 1s	C=C & C-C	284.9	59.2
		C–OH & C–O–C	285.9	19.5
		C–S	286.9	11.8
		C=O & COOH	288.0	3.2
		π–π	289.5	6.3
	N 1s	Pyridinic-N	399.1	3.1
		Pyrrolic-N	400.1	22.9
		Graphitic-N	401.0	48.4
		Oxidized-N	402.4	25.6
**GNRP**	O 1s	C=O	532.5	46.2
		C–O	533.9	49.9
		H_2O	536.8	3.9
	C 1s	C=C & C-C	285	63.6
		C–OH & C–O–C	286	15.6
		C–P	286.7	11.0
		C=O & COOH	287.9	5.4
		π–π	289.5	4.4
	N 1s	Pyridinic-N	399.4	7.9
		Pyrrolic-N	399.9	19.0
		Graphitic-N	401.1	57.6
		Oxidized-N	402.7	15.5
**GNRNS**	O 1s	C=O	532.1	40.4
		C–O	533.8	57.8
		H_2O	537.4	1.8
	C 1s	C=C & C-C	285	66.2
		C–OH & C–O–C	286	12.6
		C–S	286.7	9.5
		C–N	287.5	5.7
		C=O & COOH	288.6	2.6
		π–π	289.8	3.4

	N 1s	Pyridinic-N	398.5	17.8
		Pyrrolic-N	399.5	28
		Graphitic-N	400.6	48.6
		Oxidized-N	402.8	5.6
**GNRNP**	O 1s	C=O	532.2	41.3
		C–O	533.9	55.3
		H_2O	537.3	3.4
	C 1s	C=C & C-C	285.1	64.8
		C–OH & C–O–C	286	13.7
		C–P	286.7	7.9
		C–N	287.5	6.6
		C=O & COOH	288.7	3.1
		π–π	289.9	3.9
	N 1s	Pyridinic-N	398.9	23.1
		Pyrrolic-N	399.9	35.5
		Graphitic-N	401.2	37.5
		Oxidized-N	403.1	3.9
**GNRSP**	O 1s	C=O	532.7	43.1
		C–O	534.5	49.3
		H_2O	536.9	7.6
	C 1s	C=C & C-C	285	48.8
		C–OH & C–O–C	286.2	18.1
		C–S	287.0	10.8
		C–P	287.8	8.6
		C=O & COOH	288.8	8.8
		π–π	290.5	4.9
	N 1s	Pyridinic-N	400.3	17.2
		Pyrrolic-N	400.8	17.9
		Graphitic-N	401.5	50.2
		Oxidized-N	402.8	14.7
**GNRNSP**	O 1s	C=O	531.8	51.9
		C–O	533.8	43.4
		H_2O	536.1	4.7
	C 1s	C=C & C-C	285	48.8
		C–OH & C–O–C	286	18.1
		C–S	286.6	6.3
		C–P	287.3	7.3
		C–N	288.3	4.4
		C=O & COOH	289.4	2.2
		π–π	290.6	2.4
	N 1s	Pyridinic-N	398.7	24.2
		Pyrrolic-N	399.6	33
		Graphitic-N	400.8	37.3
		Oxidized-N	402.5	5.5

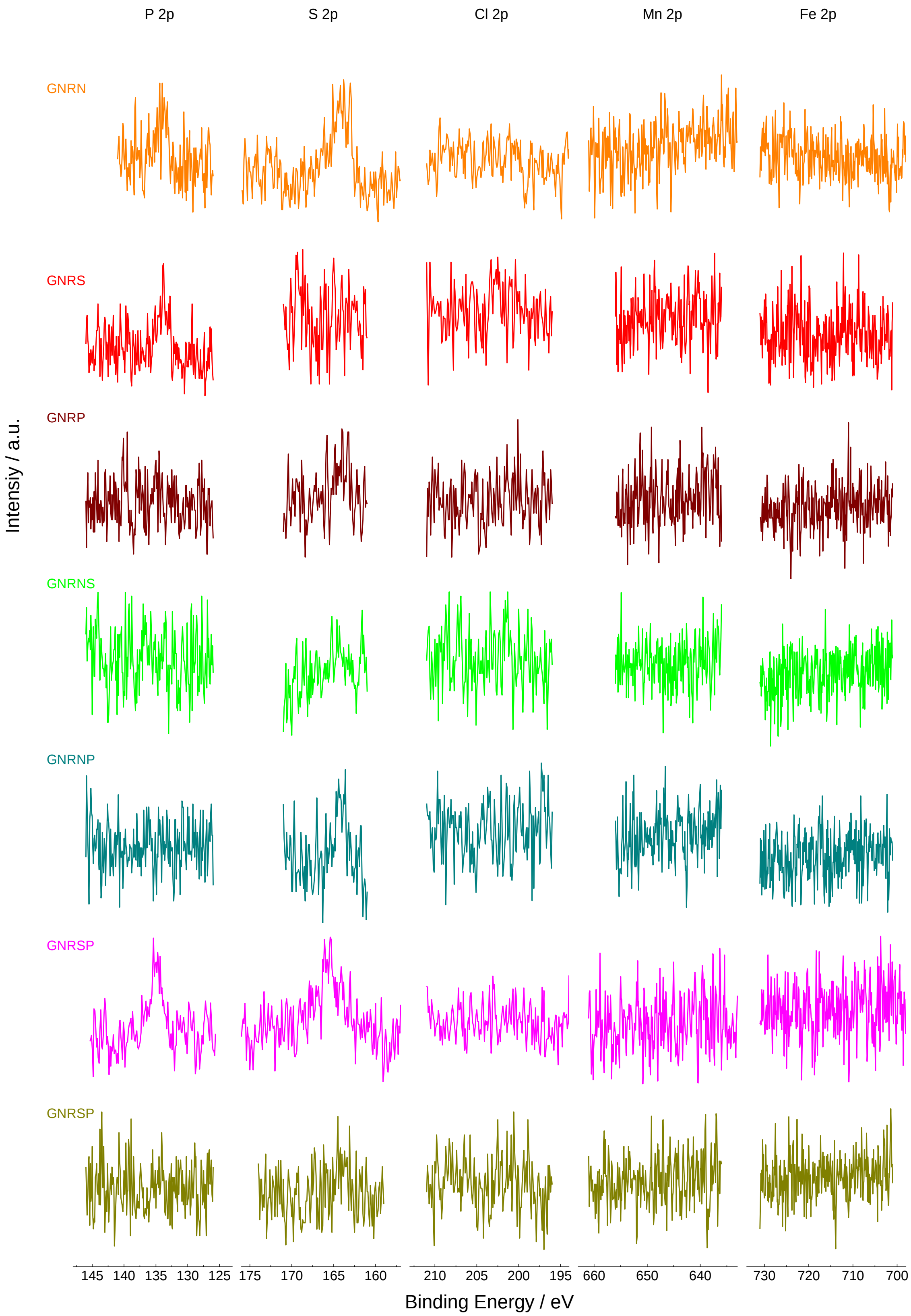

Figure S5. HR-XPS curves for the undoped and doped GNR samples.

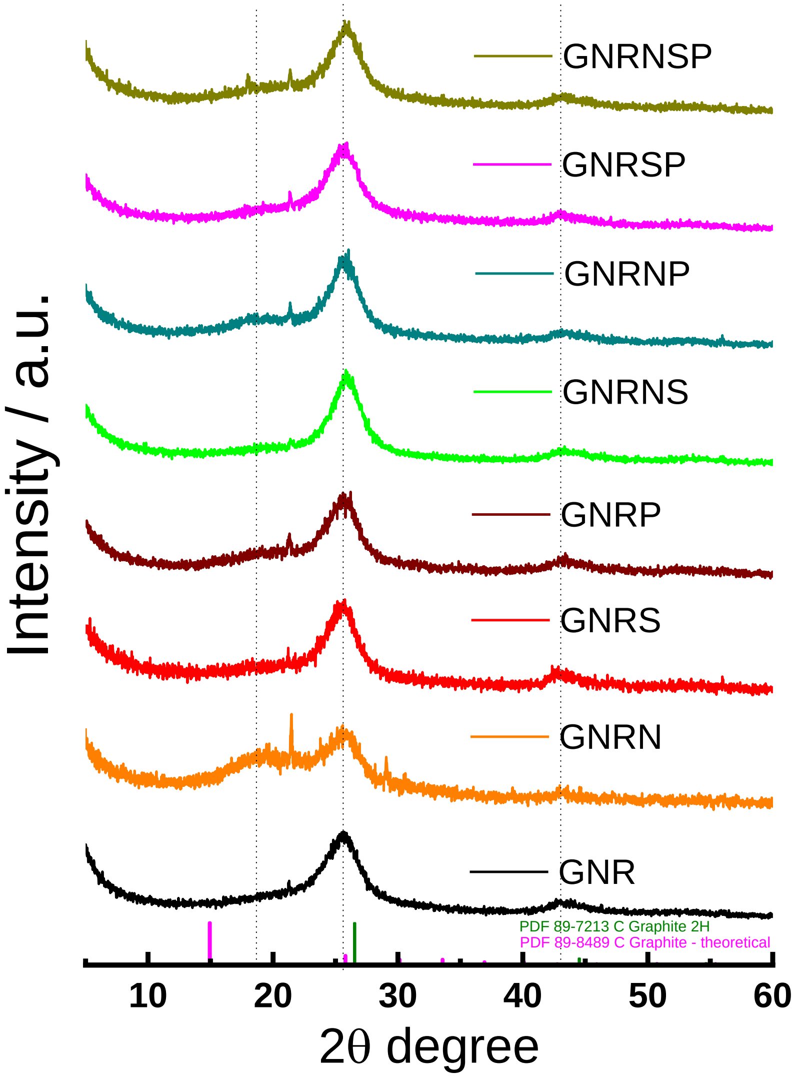

Figure S6. XRD spectra for the undoped and doped GNR samples.

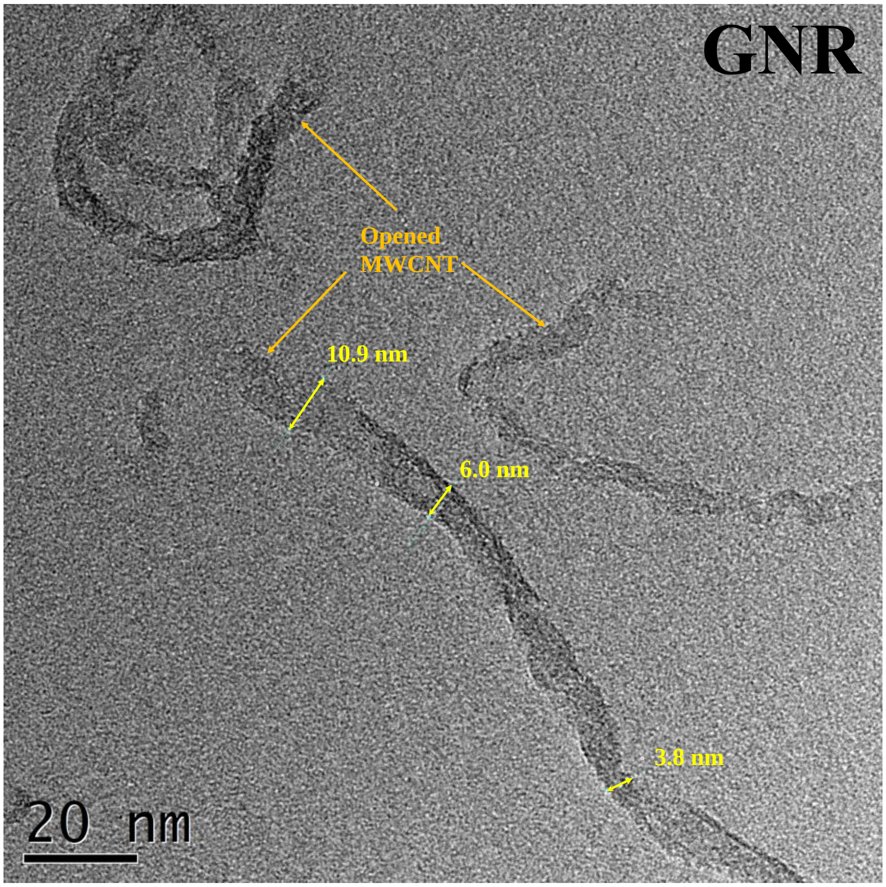
GNR
Opened
MWCNT
10.9 nm
6.0 nm
3.8 nm
20 nm

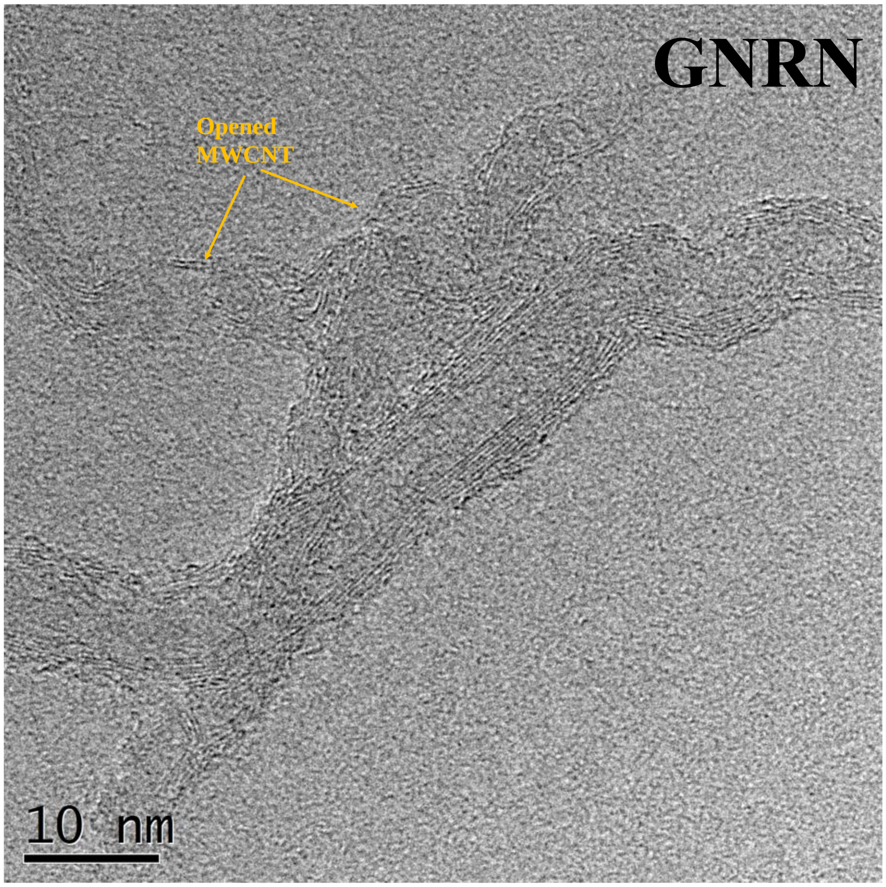
GNRN
Opened
MWCNT
10 nm

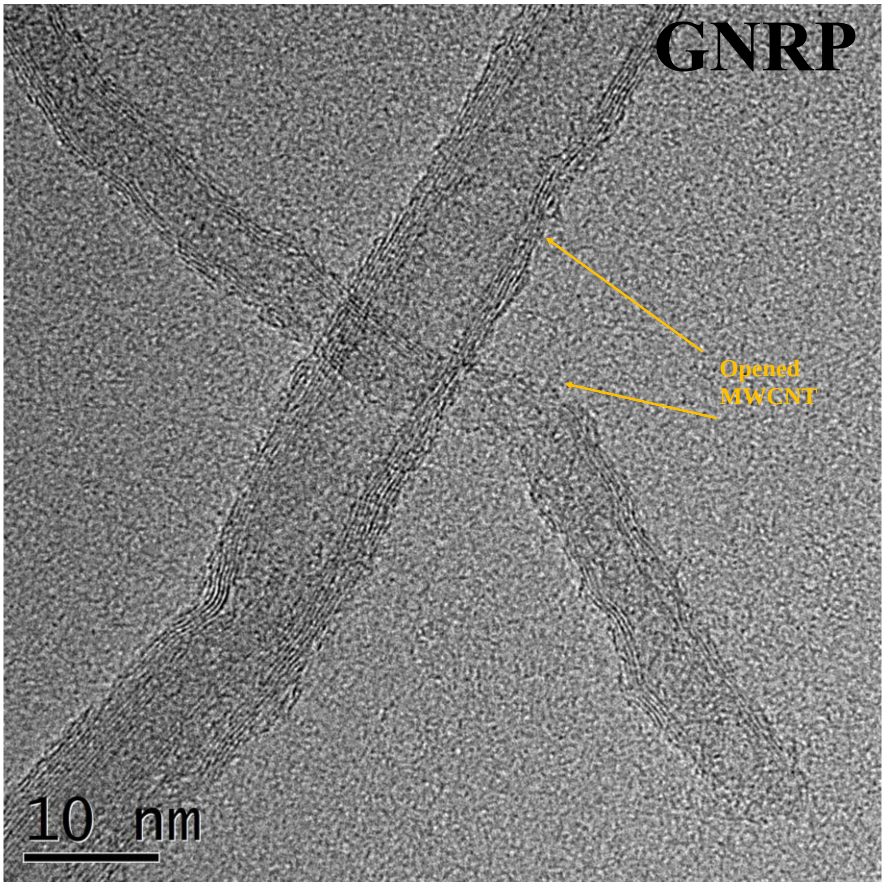
GNRP
Opened
MWCNT
10 nm

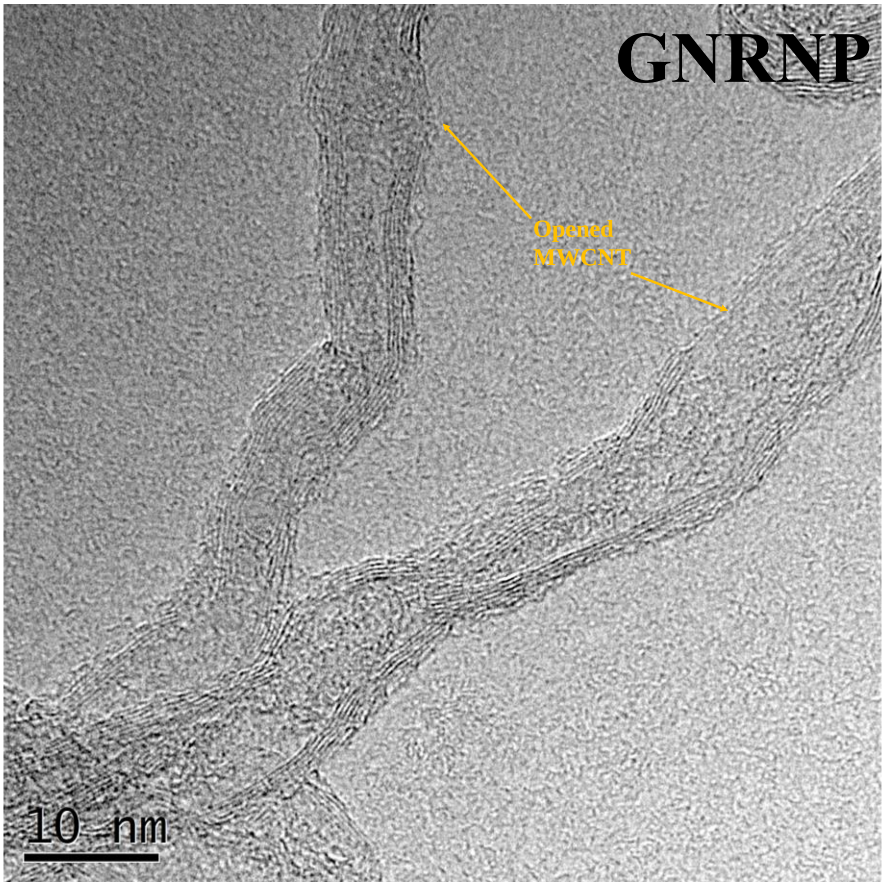
GNRNP
Opened
MWCNT
10 nm

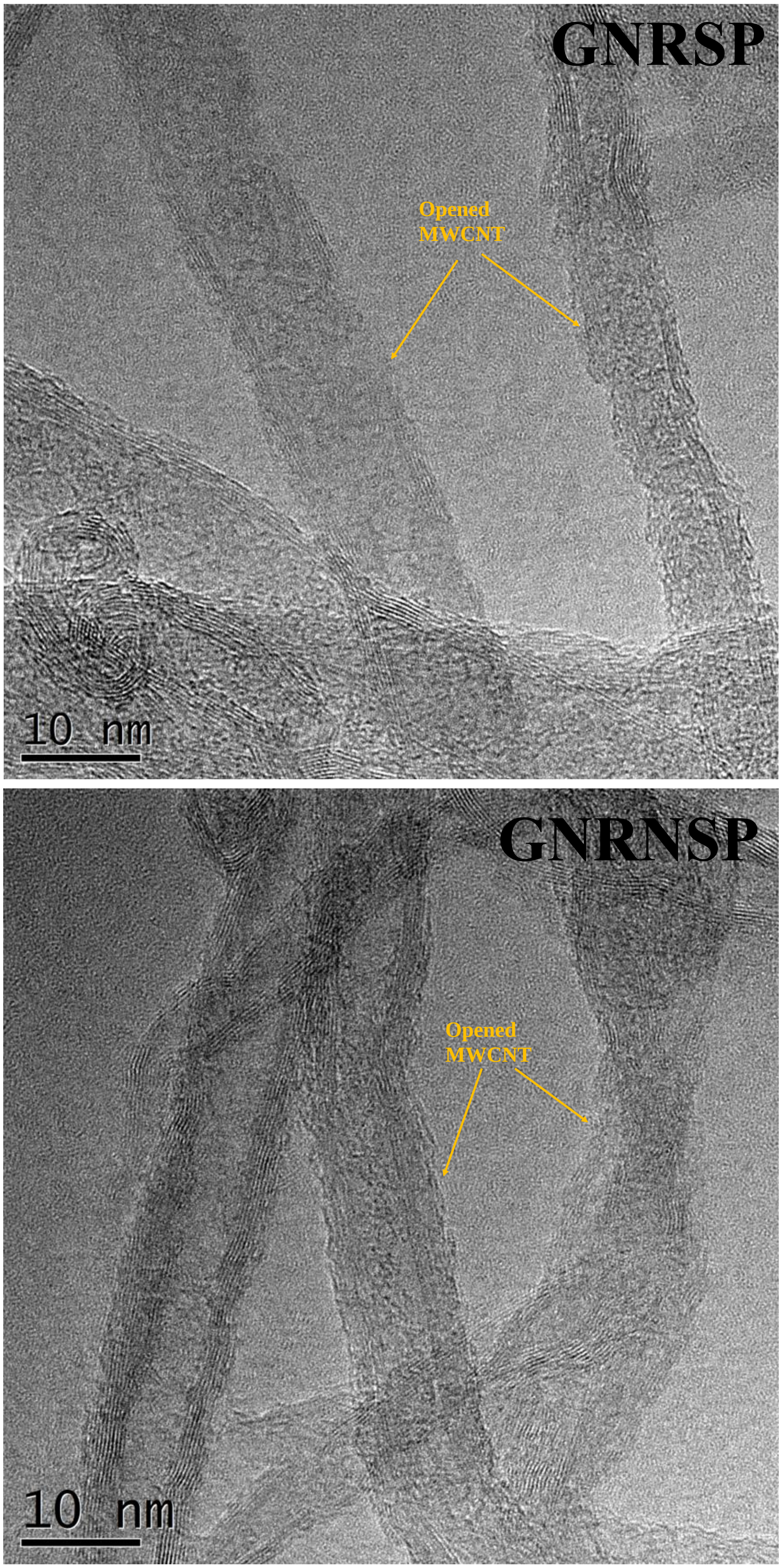

Figure S7. Representative TEM images for the undoped and doped GNR samples.

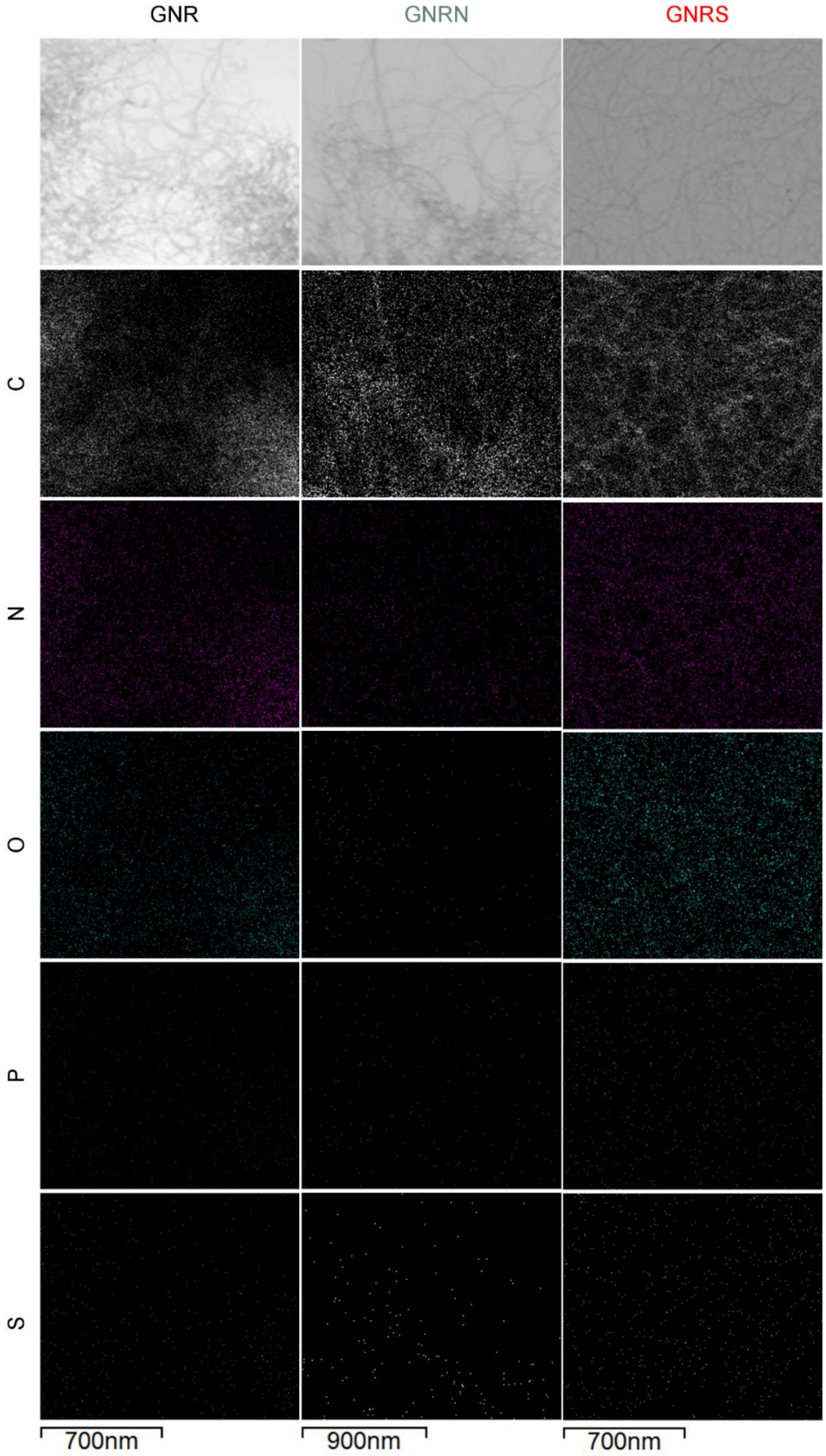

Figure S8. Scanning TEM and EDX mapping images obtained for the GNR, GNRN, and GNRS samples.

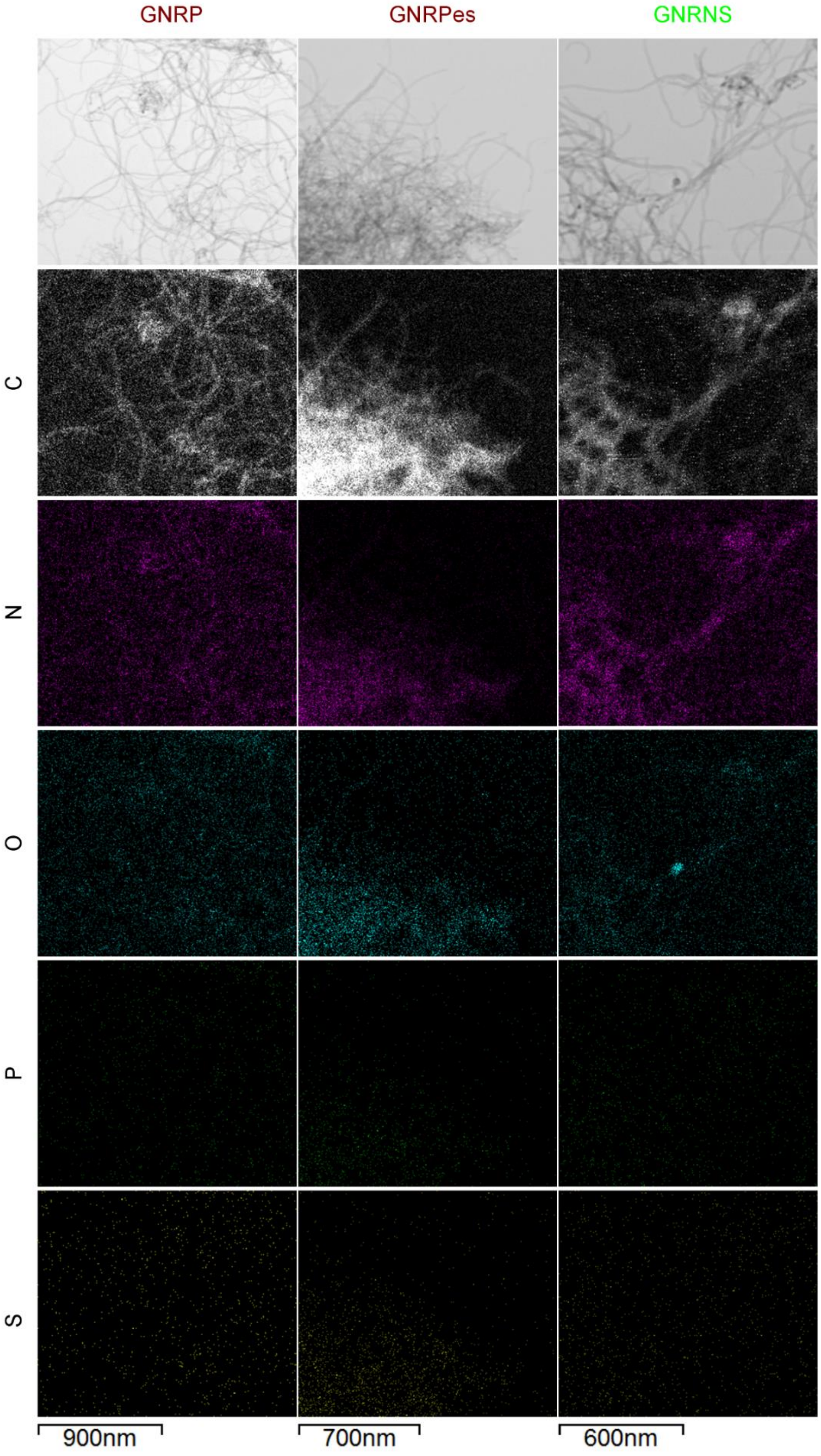

Figure S9. Scanning TEM and EDX mapping images obtained for the GNRP, GNRPes, and GNRNS samples.

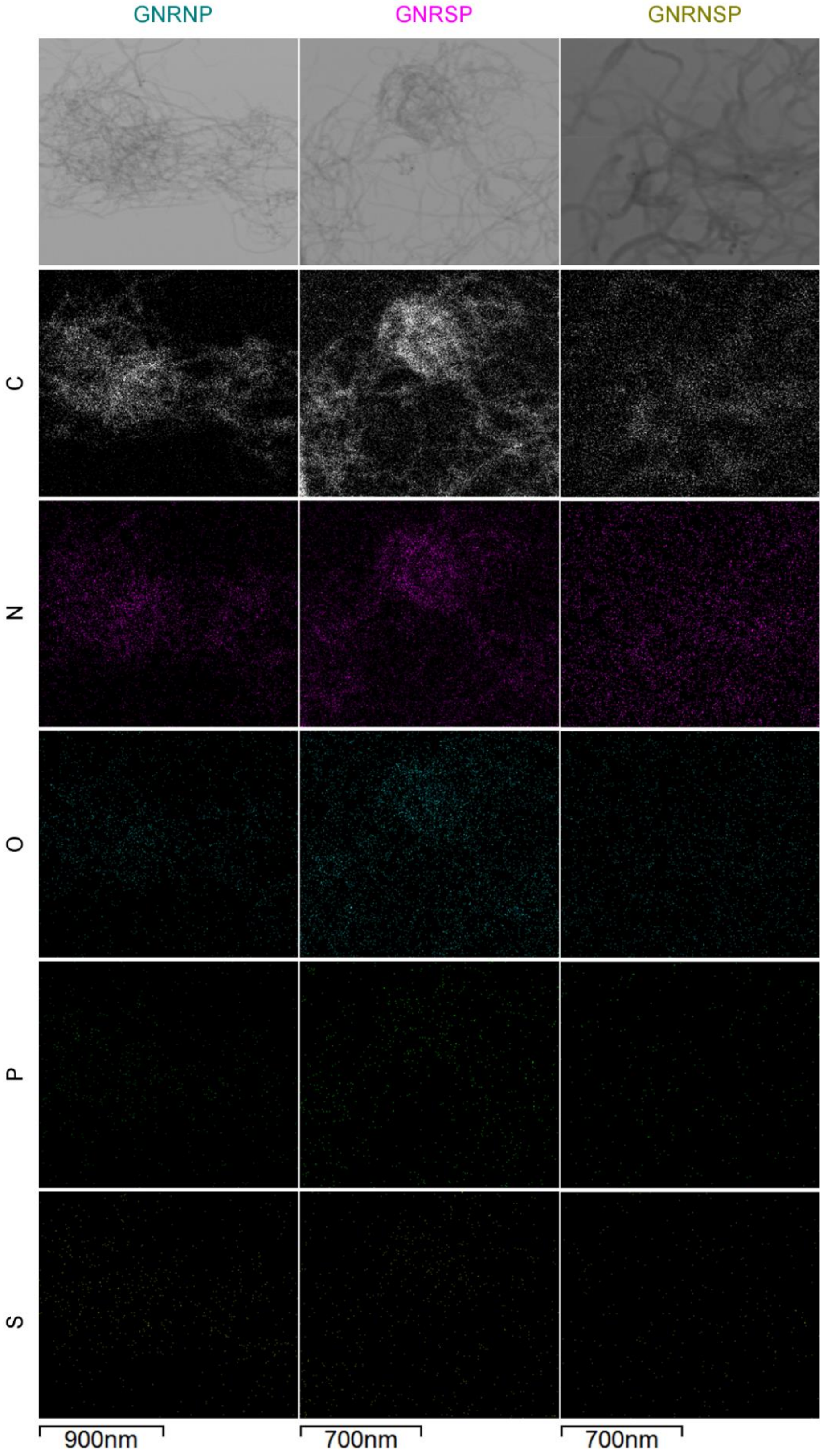

Figure 10. Scanning TEM and EDX mapping images obtained for GNRNP, GNRSP, and GNRNSP samples.

Table S6. Resistance of electrolytic solution (R_S) values obtained for the glassy carbon (GC) electrode modified with thin-film of undoped or doped GNR samples. The R_S values were obtained from the application of different N_2-saturated electrolyte solutions without electrode rotation using electrochemical impedance (data shown in Figure S5).

Catalyst film	R_S (Ω)		
	in 0.5 M H_2SO_4	*in 0.1 M K_2SO_4*	*in 0.1 M KOH*
**GNR**	4.68	71.92	36.55
**GNRN**	4.54	29.71	35.04
**GNRS**	4.44	30.58	38.82
**GNRP**	5.91	26.64	38.04
**GNRNS**	5.24	27.24	35.60
**GNRNP**	7.76	25.74	46.37
**GNRSP**	4.89	48.50	36.59
**GNRNSP**	4.46	33.69	41.00

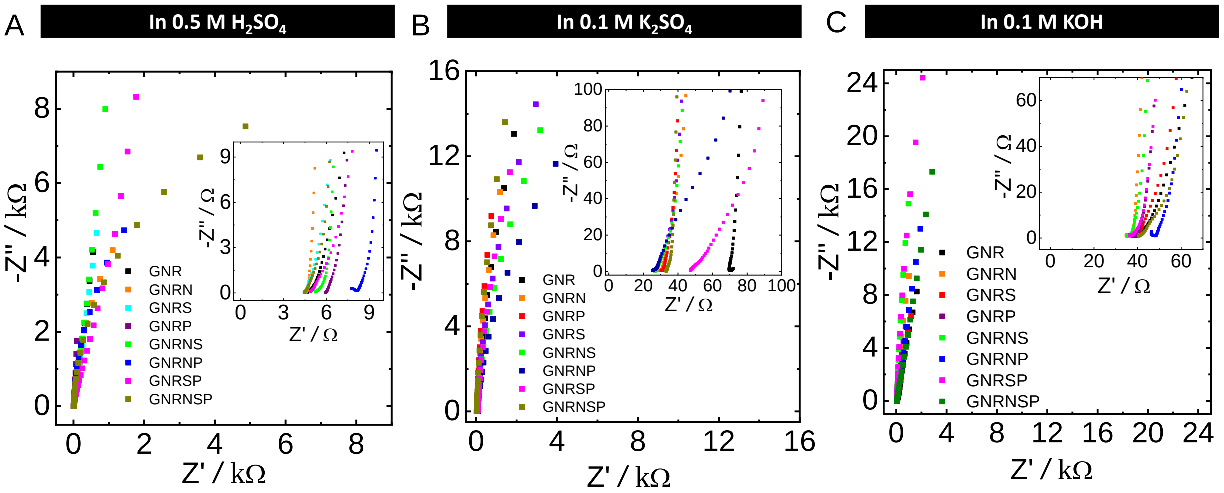

Figure S11. EIS results for the GC electrode modified with 150 μg cm^{-2} of undoped or doped GNR catalyst. The plane impedance plot was obtained in N_2–saturated (A) 0.5 M H_2SO_4, (B) 0.1 M K_2SO_4, and (C) 0.1 M KOH using the OCP value as a constant potential for the EIS measurement.

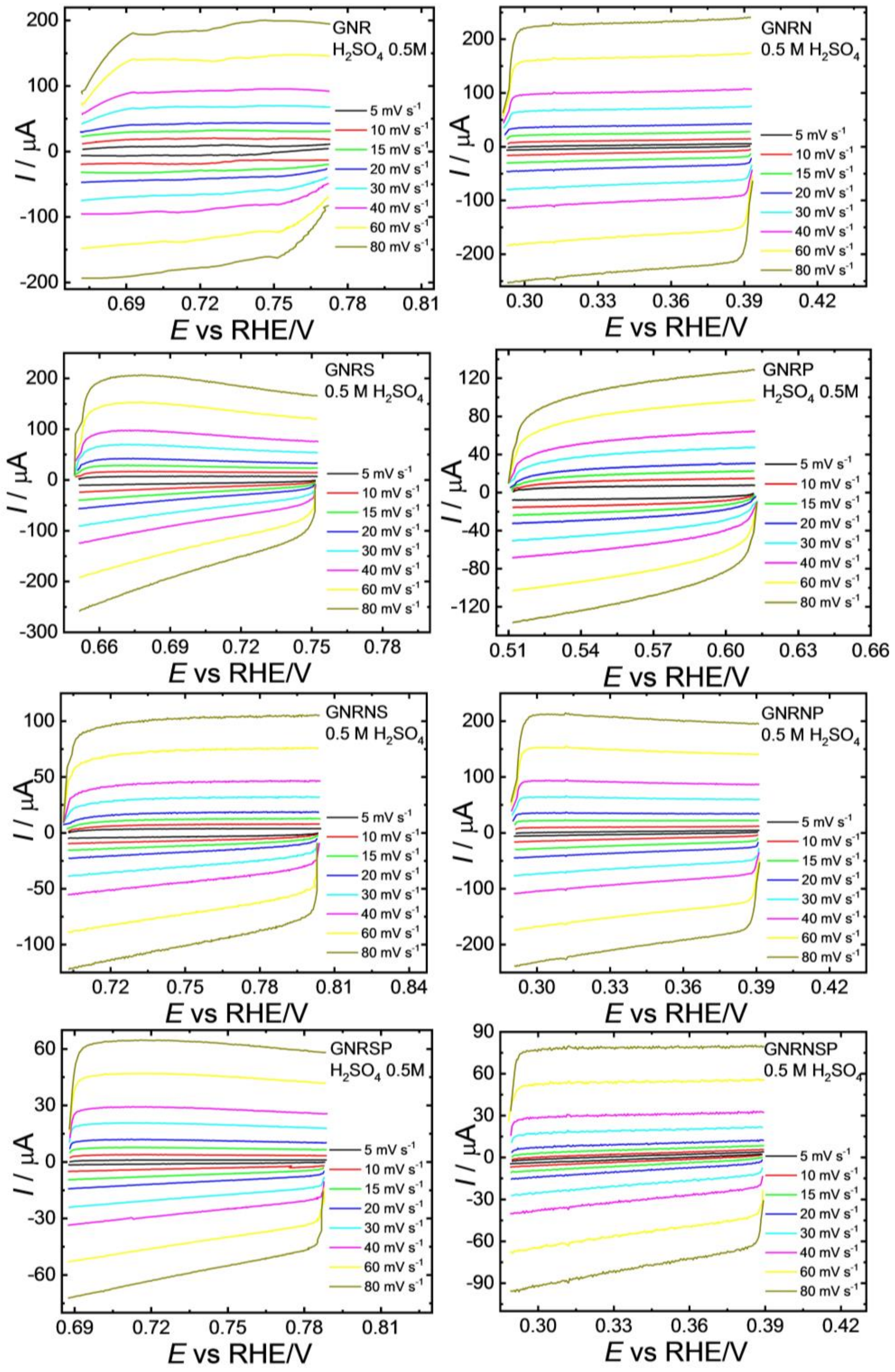

Figure S12. Cyclic voltammograms obtained in a non–Faradaic potential range for the GC electrode modified with 150 µg cm^{-2} of undoped or doped GNR catalyst in N_2–saturated 0.5 M H_2SO_4. Prior to the beginning of the subsequent potential sweep, the modified electrode was kept at each vertex potential for 10 seconds. Scanning was initiated at higher potentials.

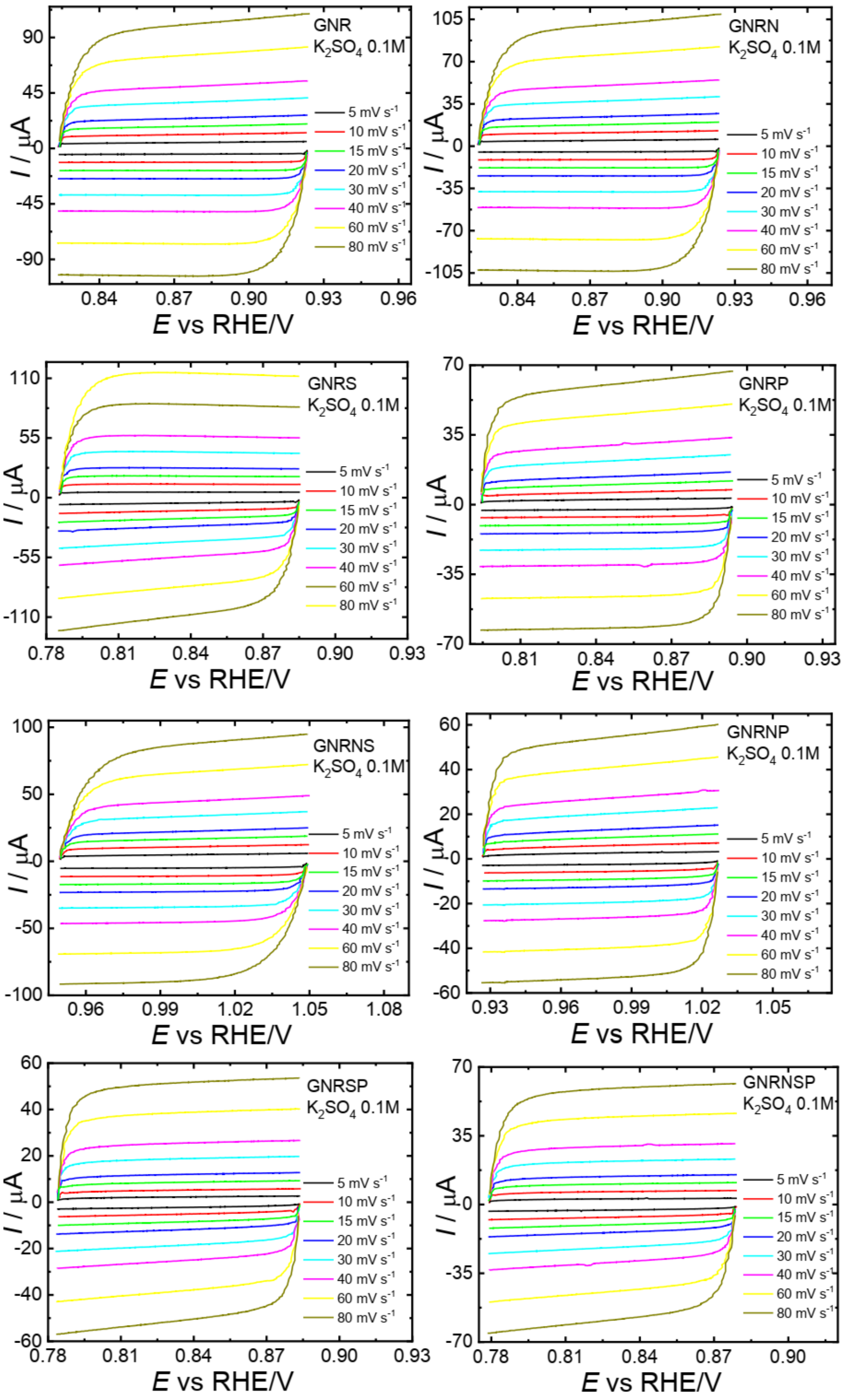

Figure S13. Cyclic voltammograms obtained in a non–Faradaic potential range for the GC electrode modified with 150 µg cm^{-2} of undoped or doped GNR catalyst in N_2–saturated 0.1 M K_2SO_4. Prior to the beginning of the subsequent potential sweep, the modified electrode was kept at each vertex potential for 10 seconds. Scanning was initiated at higher potentials.

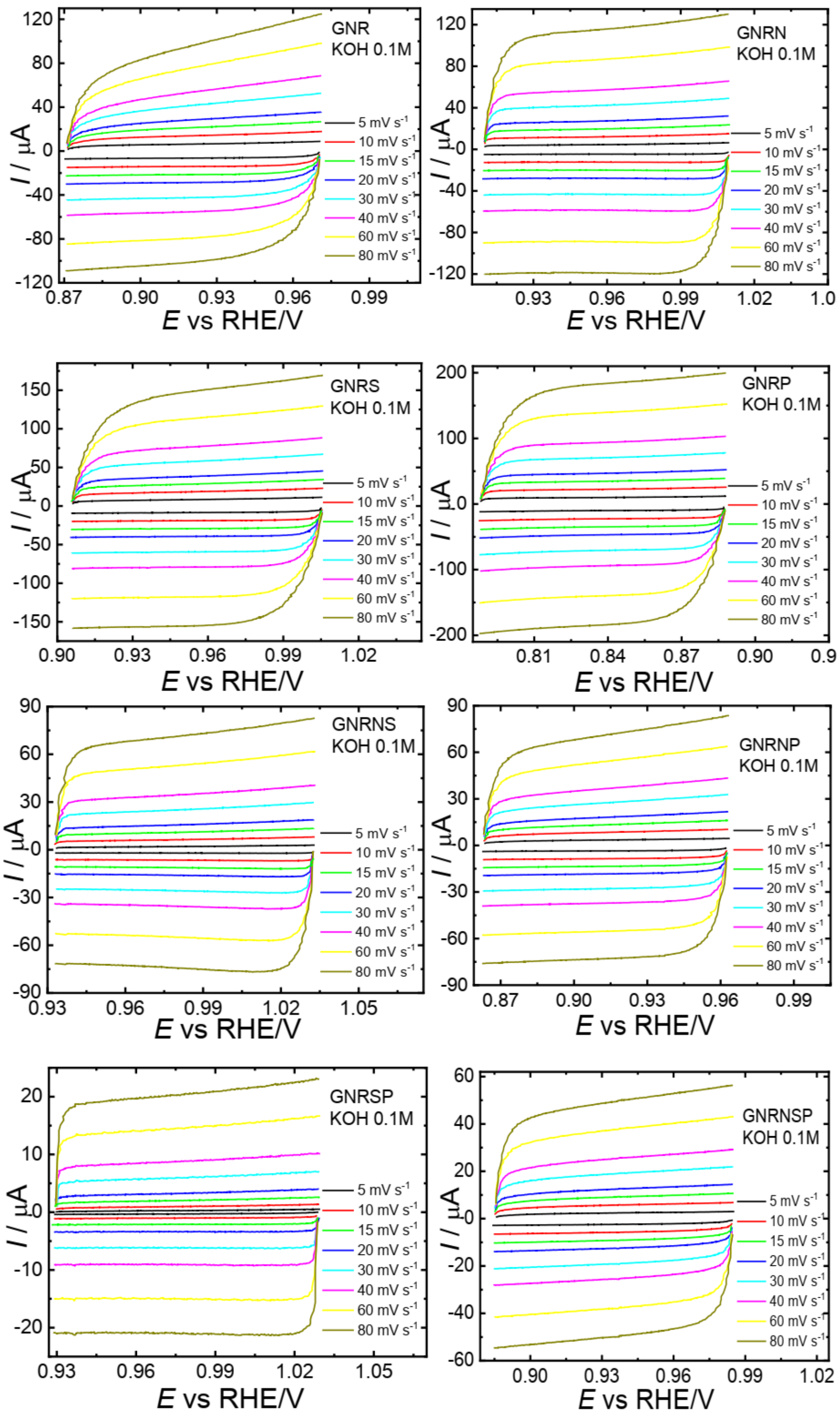

Figure S14. Cyclic voltammograms obtained in a non–Faradaic potential range for the GC electrode modified with 150 µg cm^{-2} of undoped or doped GNR catalyst in N_2–saturated 0.1 M KOH. Prior to the beginning of the subsequent potential sweep, the modified electrode was kept at each vertex potential for 10 seconds. Scanning was initiated at higher potentials.

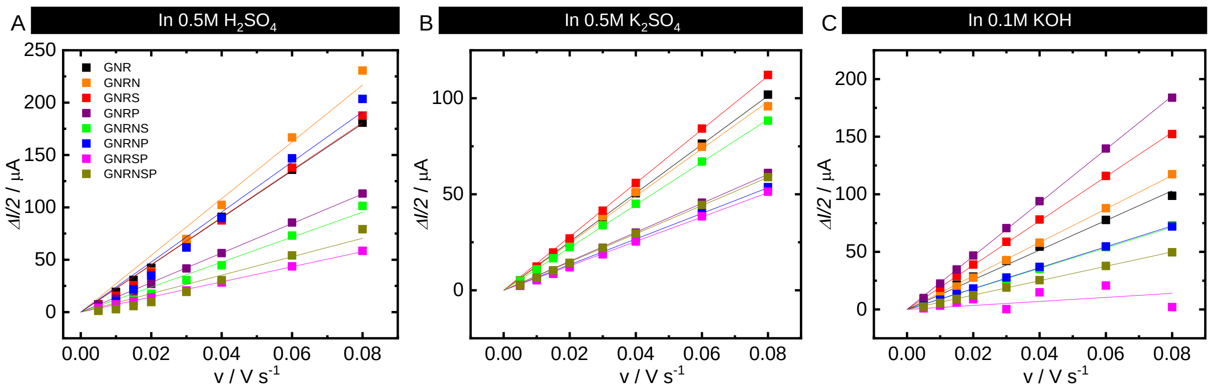

Figure S15. $\Delta I/2$ *vs.*potential scan rates (ν) plots. The plots were constructed based on the differences between anodic (I_a) and cathodic (I_c) double layer charging currents (ΔI) from (A) Figure S7 (obtained in N_2–saturated 0.5 M H_2SO_4), (B) Figure S8 (obtained in N_2–saturated 0.1 M K_2SO_4), and (C) Figure S9 (obtained in N_2–saturated 0.1 M KOH).

The selectivity toward HO_2^- production (X HO_2^-(%)) and the average number of electrons transferred per O_2 molecule (n_{av}) were calculated using the data obtained from the RRDE-based analysis (Figure 7) according with the Equations (S1) and (S2) [3]:

$$S_{\mathrm{HO_2^-}} = \frac{2I_r/N}{I_d+\frac{I_r}{N}} \text{ x } 100\% \qquad \text{(S1)}$$

where I_d is the measured disk current, I_r is the ring current, which is given by the HO_2^- oxidation, and N is the efficiency of collection for the ring electrode (N = 0.26).

$$n_{av} = \frac{4I_d}{I_d+\frac{I_r}{N}} \qquad \text{(S2)}$$

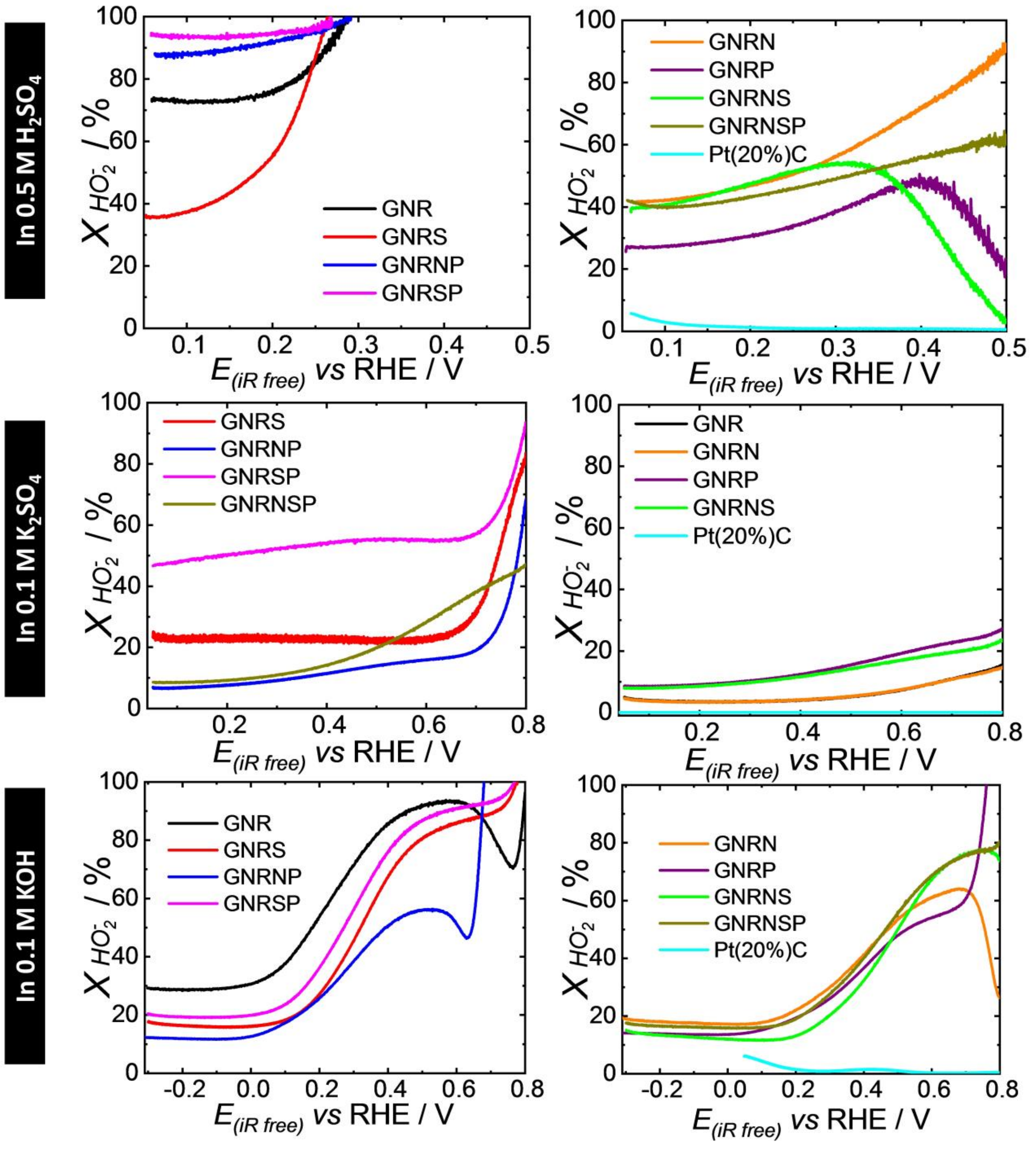

Figure S16. Selectivity for hydrogen peroxide production ($X_{H_2O_2}$) based on the electrochemical results obtained from LSV analyses conducted in a RRDE system for GC modified with 150 µg cm^{-2} of undoped or doped GNR catalyst (Figure 7) in O_2–saturated 0.5 M H_2SO_4, 0.1 M K_2SO_4, and 0.1 M KOH (1600 rpm).

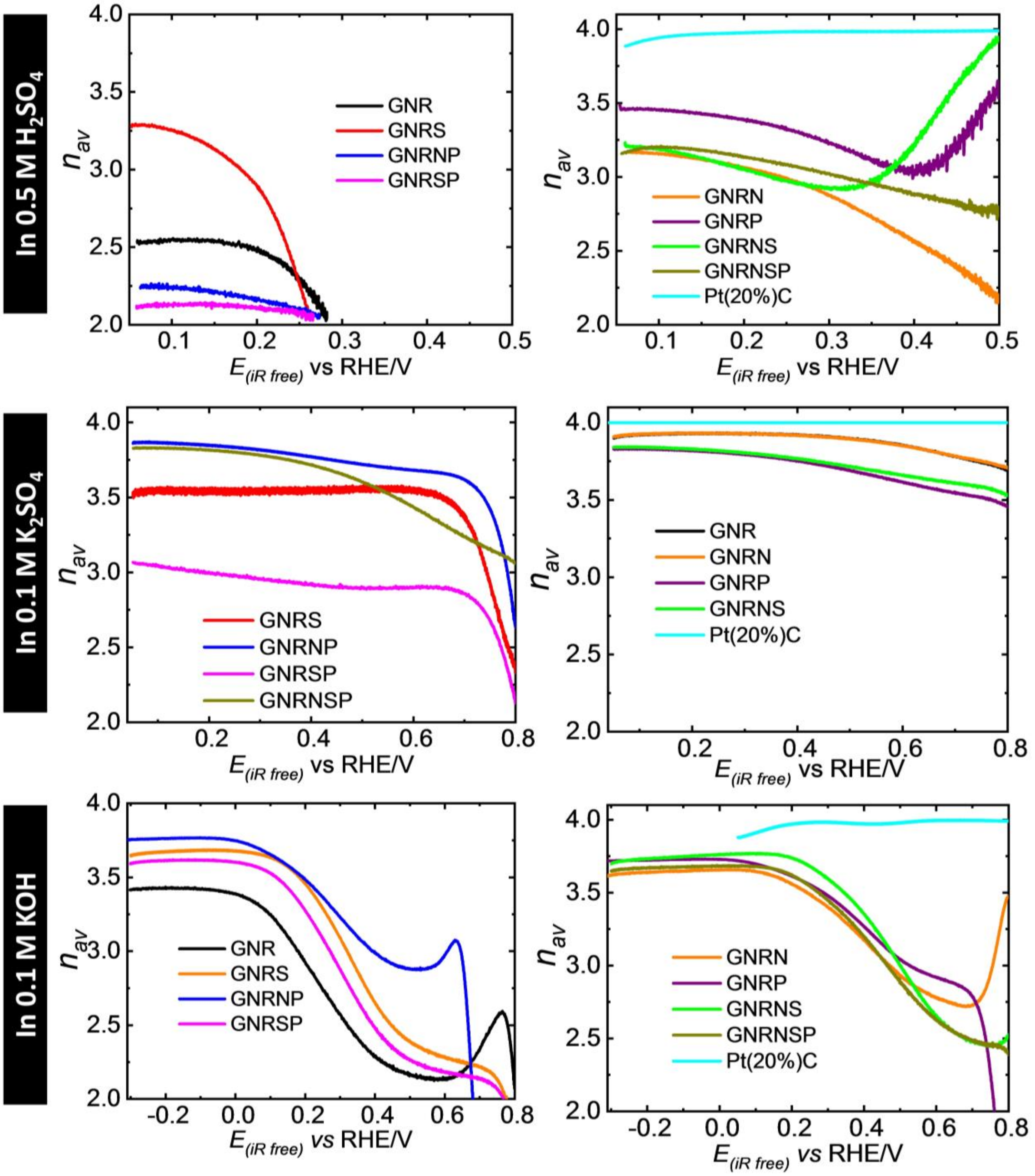

Figure S17. Average number of electrons transferred (n_{av}) during the ORR on the disk electrode at varying potentials based on the electrochemical results obtained from the LSV analyses conducted in a RRDE system for GC modified with 150 µg cm^{-2} of undoped or doped GNR catalyst (Figure 7) in O_2–saturated 0.5 M H_2SO_4, 0.1 M K_2SO_4, and 0.1 M KOH (1600 rpm).

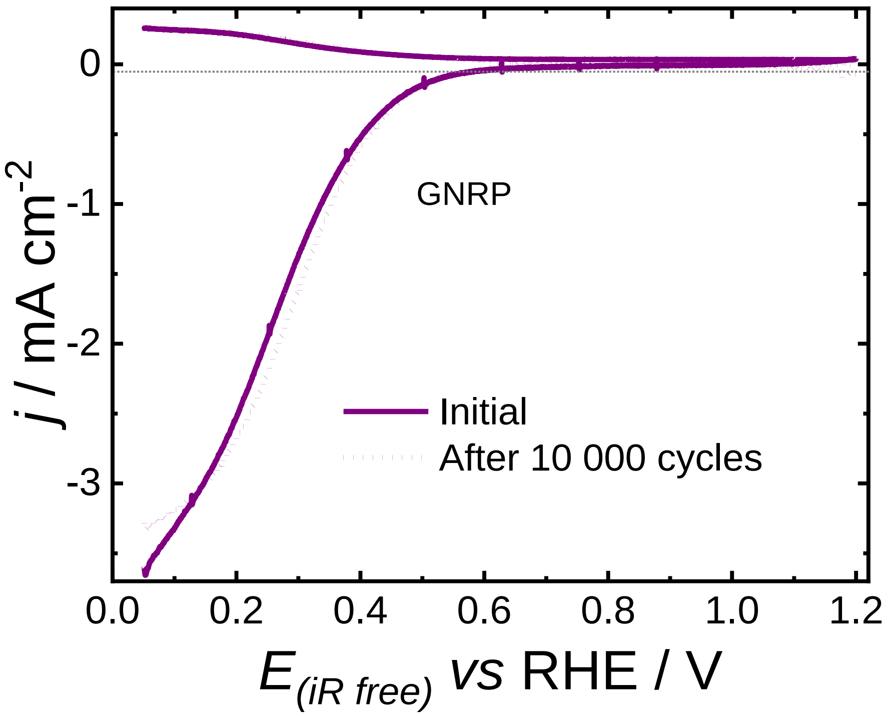

Figure S18. LSV-RRDE curves obtained in O_2–saturated 0.5 M H_2SO_4 (ω = 1600 rpm, *v* = 10 mV s^{-1}, scans were initiated at 0.05 V) for GC electrode modified with 150 µg cm^{-2} of GNRP before and after the application of 10 000 potential cycles.

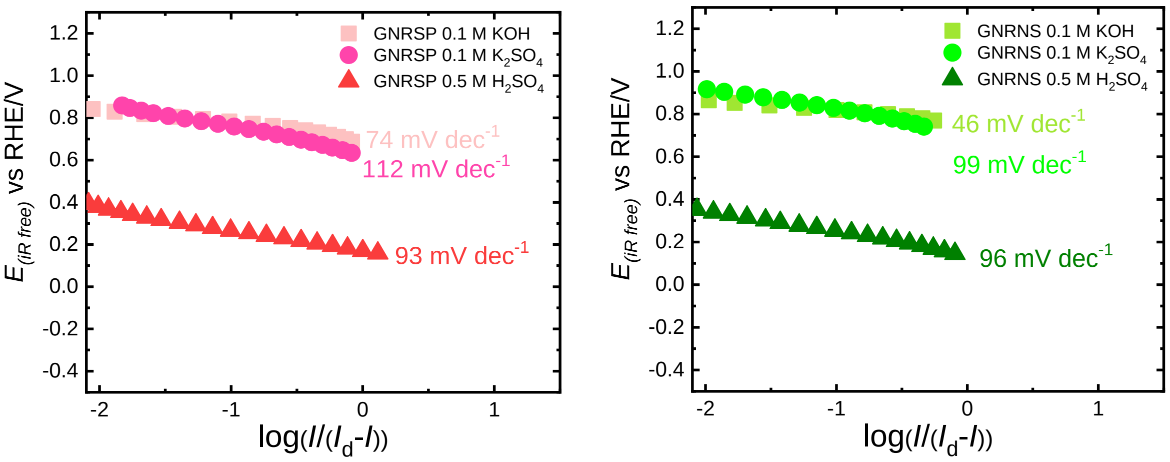

Figure S19. Tafel plots for ORR on (a) GNRSP and (b) GNRNS catalysts obtained in O_2– saturated 0.5 M H_2SO_4, 0.1 M K_2SO_4, and 0.1 M KOH electrolytes (data extracted from Figure 7).

References

[1] S.E. Colthup, N. B.; Daly, L. H.; Wiberley, Introduction to infrared and Raman spectroscopy, 3rd edn, Academic Press, Inc., 1990.

[2] R.M. SILVERSTEIN, F.X. WEBSTER, D.J. KIEMLE, Spectrometric Identification of Organic Compounds, 7th edn, John Wiley & Sons. Inc., 2005.

[3] G. V. Fortunato, L.S. Bezerra, E.S.F. Cardoso, M.S. Kronka, A.J. Santos, A.S. Greco, J.L.R. Júnior, M.R. V. Lanza, G. Maia, Using Palladium and Gold Palladium Nanoparticles Decorated with Molybdenum Oxide for Versatile Hydrogen Peroxide Electroproduction on Graphene Nanoribbons, ACS Appl. Mater. Interfaces. 14 (2022) 6777–6793. https://doi.org/10.1021/acsami.1c22362.
